# Pipeline monitoring data recovery using novel deep learning models: an engineering case study

**DOI:** 10.3389/frai.2025.1684018

**Published:** 2025-10-07

**Authors:** Yong Zhao, Xinpeng Zhang, Yanli Liu, Xuecheng Mao, Xi Chen, Yasheng Maimaitituerxun, Weidong He

**Affiliations:** ^1^Xinjiang Yaxin CBM Exploration and Development Co., Ltd., Urumqi, China; ^2^Gas Storage Co., Ltd., PetroChina Xinjiang Oilfield, Hutubi, China; ^3^School of Civil Engineering and Geomatics, Southwest Petroleum University, Chengdu, China

**Keywords:** data recovery, pipeline monitoring, optical-fiber sensing, prairie dog optimization, deep learning

## Abstract

Pipeline monitoring frequently encounters missing data, leading to incomplete evaluation and hindering a comprehensive assessment of the pipeline’s structural health. To address this issue, this study proposes a novel PDO-BiGRU-GAN model for missing data recovery. The model integrates three components: the prairie dog optimization algorithm (PDO) for hyperparameter tuning, the bidirectional gated recurrent unit (BiGRU) for effective temporal feature extraction, and the generative adversarial network (GAN) for data generation and completion. A comprehensive monitoring database was established using field data from an open-source pipeline project. The contributions of individual modules to the overall performance were evaluated via hyperparameter sensitivity analysis and ablation studies. The impact of missing data ratio and the number of missing sensors on the model’s recovery performance was analyzed. In addition, the proposed model was compared with eight existing mainstream deep learning models. The results show that each component of the PDO-BiGRU-GAN significantly enhances overall performance. The model achieves strong recovery accuracy across various missing data scenarios, with the R^2^ consistently exceeding 0.93. Moreover, the model performs optimally when the missing data ratio is below 20/24. Compared to other models, PDO-BiGRU-GAN achieves the highest R^2^ and the lowest error metrics (MSE, RMSE, MAPE, MAE). In terms of computational efficiency, the model requires slightly more processing time than simpler models but is faster than more complex models. Overall, the proposed model provides a robust and scalable solution for pipeline monitoring data recovery, advancing intelligent pipeline health assessment and supporting the development of infrastructure safety management and smart monitoring technologies.

## 1 Introduction

Pipeline systems are essential infrastructure supporting multiple urban functions such as water supply, drainage, heating and gas distribution (Mazumder et al., 2018; Xiong et al., 2020). Their safe operation is vital for ensuring stable urban systems and sustainable daily life. However, pipelines are typically laid across regions, over long distances, and beneath the ground (Li et al., 2022; Quej-Ake et al., 2020; Shirazi et al., 2023). They often traverse diverse geological formations and complex subsurface terrains. During service, pipelines are prone to structural damage due to geostress variations, soil-induced degradation, and hydrochemical reactions, potentially leading to deformation and leakage (Sharma et al., 2024; Wong and McCann, 2021). To address these challenges, a comprehensive system spanning the entire lifecycle of pipeline infrastructure is necessary for monitoring and assessment.

Commonly used pipeline monitoring techniques include acoustic detection, fiber-optic sensing, electromagnetic induction, pressure monitoring, and image recognition (Adegboye et al., 2019; Ho et al., 2020; Sun et al., 2025a). Acoustic detection offers rapid response and is effective for leak localization; however, it is highly susceptible to environmental noise and interference (Xu et al., 2013). Electromagnetic induction is suitable for detecting corrosion in metallic pipelines and benefits from non-contact operation, but it is limited to conductive materials and suffers from rapid signal attenuation (Vasagar et al., 2024). Pressure monitoring is structurally simple and cost-effective, providing basic insights into operational conditions, though it lacks multidimensional data and cannot effectively identify structural damage (Ciang et al., 2008; Lopez and Sarigul-Klijn, 2010). Image recognition enables intuitive visualization but relies on favorable environmental conditions and has limited coverage over large areas (Cai et al., 2019; Zhou, 2023). In contrast, fiber-optic sensing provides high sensitivity, supports long-distance continuous monitoring, and is relatively cost-effective (Sun et al., 2025b). Therefore, fiber-optic sensing systems offer significant potential for widespread application in pipeline monitoring.

However, similar to traditional electrical sensors, fiber-optic sensing technology still faces data loss (Feng et al., 2019). First, signal interruptions may occur at sensor splicing points due to manufacturing defects or mechanical stress (Kuntoğlu et al., 2021). Second, prolonged use can cause sensor material aging, resulting in failure to acquire valid data. Additionally, external factors such as geological shifts, construction disturbances, and chemical corrosion can damage sensors, affecting data continuity and accuracy (Wright et al., 2019). Moreover, fiber-optic systems typically require long-distance signal transmission, during which signal attenuation at splicing points accumulates along the transmission path and may result in the loss or distortion of valid data (Sun et al., 2025c). Furthermore, environmental noise can obscure or disrupt the original measurement signals, causing further loss of valid information.

Data loss undermines the monitoring system’s ability to perceive critical operational states in real time, compromises data integrity, and reduces analytical accuracy (Lei et al., 2023; Tan et al., 2016). Moreover, it increases the response delay and uncertainty in fault detection. In severe cases, data loss may obscure early risk indicators, weakening the system’s warning capability (Avula, 2021; Jieyang et al., 2023). Furthermore, prolonged data gaps can lead to insufficient accumulation of historical data, impairing trend analysis of pipeline structural performance and lifespan prediction. Consequently, this affects the scientific basis for pipeline safety assessment and management strategy development. Therefore, it is imperative to develop effective solutions addressing data loss in fiber-optic sensing systems.

To address data loss issues, deep learning provides effective technical solutions (Bao et al., 2025; Li et al., 2025a; Ressi et al., 2022, 2024a; Sun et al., 2024a). Deep learning is a machine learning approach based on artificial neural networks, whose core concept is inspired by the structure of the human brain (Liu et al., 2025a; Sun et al., 2024b; Wang et al., 2025a, 2025b, 2025c). It employs multiple layers of nonlinear transformations to automatically extract complex features from data, enabling efficient modeling of large-scale information (Wei et al., 2025). Compared to traditional machine learning, deep learning automatically captures intricate nonlinear relationships within data, thereby reducing reliance on manual feature engineering (Sun et al., 2024c). In the field of data recovery, deep learning leverages spatiotemporal dependencies in incomplete datasets to reconstruct missing information with high accuracy (Lei et al., 2021). Common models for data loss recovery include the standard GAN (Sun et al., 2025c), LSTM-GAN (Pu et al., 2022; Kumari et al., 2024), GRU-GAN (Huang et al., 2022; Shen et al., 2022), Bi-LSTM-GAN (Jiang et al., 2023), and STOA-Bi-LSTM-GAN (Sun et al., 2025c), all of which have demonstrated promising effectiveness in preliminary applications.

Nevertheless, existing models still exhibit several limitations. When applied to time-series data, the standard GAN often suffers from unstable training of the generator and discriminator. It fails to effectively capture sequential dependencies, resulting in imputed values that deviate from actual dynamic patterns (Ren and Xu, 2019). LSTM-GAN and GRU-GAN alleviate this issue by employing recurrent neural networks (LSTM or GRU), thereby improving the ability to model long-term dependencies. However, these models remain vulnerable to vanishing or exploding gradients when processing multivariate or long-sequence data. Their performance is also highly sensitive to hyperparameter settings, such that minor deviations can lead to overfitting or unstable generation (Wan and Liu, 2023). BiLSTM-GAN enhances modeling by leveraging both past and future contextual information, yet it is still prone to mode collapse, unstable convergence, and challenging hyperparameter optimization (Wan, et al., 2023). STOA-BiLSTM-GAN demonstrates strong results across multiple benchmark datasets, but its high complexity and intensive training requirements hinder scalability in large-scale industrial applications. Therefore, there is an urgent need for a novel deep learning framework that maintains recovery accuracy while reducing model complexity and computational cost, thereby enhancing practicality and scalability.

Based on this, the study proposes a novel PDO-BiGRU-GAN model that integrates the prairie dog optimization algorithm (PDO), bidirectional gated recurrent units (BiGRU), and generative adversarial network (GAN). This model is designed to recover missing fiber-optic sensing data. Monitoring data from an open-source pipeline project were used to construct a pipeline monitoring dataset. Hyperparameter sensitivity analysis and ablation experiments were performed to evaluate the necessity and contribution of each module. The impact of ratios of missing data and the number of missing sensors on the recovery performance of the PDO-BiGRU-GAN model was analyzed. Furthermore, the model’s accuracy and computational efficiency were compared with eight existing deep learning models.

The main contributions and innovations of this study are as follows: First, a novel PDO-BiGRU-GAN deep learning framework was developed. This framework integrates the hyperparameter optimization capability of the PDO module, the temporal feature extraction capability of the BiGRU module, and the data generation and imputation ability of the GAN module. Second, fiber-optic monitoring data were obtained from an open-access pipeline project, and the model was systematically evaluated through hyperparameter sensitivity analysis and ablation studies. Additionally, the proposed model was compared with eight existing mainstream models in terms of accuracy and computational efficiency under varying missing-data scenarios. Overall, the proposed framework provides a new approach for imputing missing pipeline monitoring data and plays a crucial role in ensuring the completeness of pipeline monitoring information.

## 2 Motivation for developing the PDO-BiGRU-GAN network

To address the issue of missing monitoring data, numerous studies have focused on developing techniques for managing missing values in time-series data. Traditional approaches can be broadly categorized into two types. The first involves case deletion, which removes observations containing missing values to avoid their impact on analytical results (Schafer and Graham, 2002; Silva and Zárate, 2014). The second includes statistical methods, such as spline interpolation, matrix completion, and mean imputation, which replace missing data points based on statistical estimates (Gu et al., 2021). However, these methods have notable limitations. First, they exhibit limited capability in modeling temporal dependencies and often fail to capture complex dynamic relationships among variables. Second, their performance becomes unstable under conditions with a high missing ratio, leading to substantial errors (Zhang and Wang, 2024). Finally, they struggle to accurately reconstruct the underlying data distribution, particularly when the interactions among multiple features are intricate (Zhang and Wang, 2024). Consequently, traditional methods often fail to meet the accuracy and reliability requirements for reconstructing pipeline monitoring data.

In recent years, researchers have explored the use of GANs to recover missing data. Unlike traditional methods, GANs can model the underlying data distribution, thereby enabling high-quality data imputation. This capability enhances the completeness and reliability of the recovered data. The advantages of GANs are particularly pronounced in multivariate time-series analysis. They can not only impute individual missing values but also maintain complex dependencies among variables (Oh et al., 2021). Moreover, the generative framework of GANs enables adaptation to diverse missing data patterns, improving the robustness and flexibility of recovery outcomes (Gong et al., 2022). Studies have demonstrated that GANs achieve high accuracy in handling missing data, providing strong support for data analysis, prediction, and decision-making across various domains (Kachuee et al., 2020).

GANs have been widely applied to data recovery tasks. However, existing GAN-based approaches often neglect the interrelationships among variables and the bidirectional temporal dependencies inherent in time-series data (Wu et al., 2021). An ideal time-series imputation method should capture both characteristics while accurately modeling the underlying data distribution. Integrating temporal feature extraction modules into the GAN framework can therefore enhance model performance. Among temporal feature extraction networks, recurrent neural networks (RNNs) are particularly well-suited for modeling complex temporal dependencies. Nevertheless, they exhibit inherent limitations, including a restricted ability to extract fine-grained information and susceptibility to gradient explosion and vanishing. These issues reduce training efficiency and limit their applicability in temporal feature extraction tasks (Li et al., 2024). Recent advances in RNN architectures have addressed some of these challenges. In particular, the development of GRUs mitigates the vanishing gradient problem, accelerates convergence, and reduces computational uncertainty (Noh, 2021). Building on this, BiGRUs were introduced to process sequences in both forward and backward directions. Compared with BiLSTMs, BiGRUs retain the ability to model long-term dependencies. They also maintain a simpler structure, have fewer parameters, offer higher computational efficiency, and enable more stable training. They also demonstrate reduced sensitivity to overfitting in small datasets or noisy environments. BiGRUs excel at capturing both local temporal patterns and global trends, effectively lowering predictive uncertainty and alleviating forgetting effects. Therefore, embedding a BiGRU module within a GAN framework leverages the generative adversarial mechanism to approximate the original data distribution. It also fully exploits bidirectional temporal dependencies for feature modeling. This approach achieves higher accuracy and more robust performance in time-series missing value recovery tasks.

Furthermore, the performance of the BiGRU-GAN network is strongly influenced by hyperparameter configurations (Richter et al., 2024). Existing studies typically rely on manual trial-and-error to identify optimal parameter combinations, a process that is both inefficient and prone to producing suboptimal predictive results. Consequently, the use of advanced optimization algorithms for automated hyperparameter search is essential. Inspired by prairie dogs’ natural behaviors, Ezugwu et al. (2022) proposed the PDO algorithm. They compared PDO with several classical optimization methods, including the arithmetic optimization algorithm (Abualigah et al., 2021), grey wolf optimizer (Mirjalili et al., 2014; Sun et al., 2024d), differential evolution optimizer (Kosorukoff, 2001), salp swarm optimizer (Mirjalili et al., 2017), biogeography-based optimizer (Simon, 2008), sine cosine optimizer (Mirjalili, 2016), particle swarm optimizer (Kennedy and Eberhart, 1995; Sun et al., 2023a), and dwarf mongoose optimizer (Agushaka et al., 2022). Experimental results demonstrate that PDO excels in searching for the global optimum and exhibits a more stable convergence process than many of these algorithms. Statistical analyses further confirm its robustness in balancing exploration and exploitation. By leveraging these advantages, PDO can be integrated into the BiGRU-GAN framework to significantly improve hyperparameter search efficiency, thereby enhancing predictive accuracy and model stability. Based on this approach, the present study develops a PDO-BiGRU-GAN network to better capture the spatiotemporal correlations between missing and available data.

## 3 Basic principles of the PDO-BiGRU-GAN network

This study proposes a novel hybrid model, PDO-BiGRU-GAN, for missing data imputation. This model consists of three components: the PDO module, the BiGRU module, and the GAN module. The BiGRU module models the bidirectional temporal dependencies between available and missing data, thereby enhancing the representation of time series information (Du et al., 2019). The PDO module optimizes critical hyperparameters of the BiGRU model (learning rate, batch size, units per layer, and number of layers) to improve training efficiency and generalization performance. The GAN module introduces an adversarial mechanism to further enhance the realism and distribution consistency of the recovered data. The following sections provide a detailed explanation of the principles underlying each module of the PDO-BiGRU-GAN network.

### 3.1 BiGRU module

The GRU is an improved variant derived from the long short-term memory (LSTM) network. It merges the forget gate and the input gate of the LSTM into a single update gate (Richter et al., 2024). By integrating the cell state and hidden state of the LSTM, the GRU effectively mitigates the vanishing and exploding gradient problems encountered in modeling long-term dependencies. Furthermore, this architecture enhances both the convergence speed and computational efficiency of the model. The GRU primarily consists of two gates: the reset gate and the update gate, which, respectively, regulate the forgetting and retention of information before and after data transmission. This mechanism enables effective control and propagation of information within the neural units. The computational formulas of the GRU are presented in Equation 1.


(1) $$\left\{ \begin{array}{c} z_{t}=\sigma (W_{Z}\cdot [h_{t-1},x_{t}]+b_{z})\\ r_{t}=\sigma (W_{r}\cdot [h_{t-1},x_{t}]+b_{r})\\ {\tilde{h}}_{t}=\tanh(W_{r}\cdot [r⊙h_{t-1},x_{t}]+b_{h})\\ h_{t}=(1-z_{t})⊙h_{t-1}+z_{t}⊙{\tilde{h}}_{t} \end{array}\right.$$

Where *r_t_* denotes the reset gate; *Z_t_* represents the update gate; *h_t_* is the output; ${\tilde{h}}_{t}$ is the candidate activation; and *σ* denotes the sigmoid activation function.

**Figure 1:**
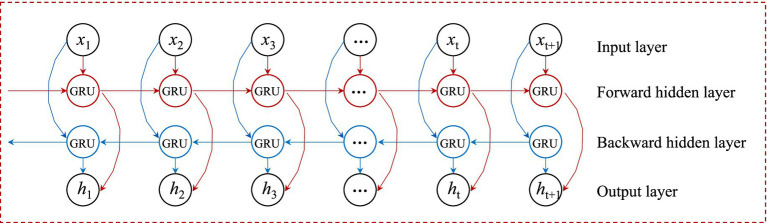
Structure diagram of the BiGRU model.

BiGRU is an extension of the GRU model that integrates information flow from both forward and backward directions, thereby enhancing its ability to process time series data. By simultaneously capturing dependencies in both directions, the model achieves a more comprehensive understanding of the dynamic patterns within time series. Compared to the unidirectional GRU, BiGRU more effectively captures intricate temporal features, significantly improving prediction accuracy. This model maximizes the extraction of key features relevant to forecasting, resulting in enhanced precision and stability. The computational formulas of the model are shown in Equation 2. A schematic diagram of the BiGRU architecture is presented in Figure 1.


(2) $$\left\{ \begin{array}{l} {\vec{h}}_{t}=\mathrm{GRU}(x_{t},{\vec{h}}_{t-1})\\ {\overset{\leftarrow }{h}}_{t}=\mathrm{GRU}(x_{t},{\overset{\leftarrow }{h}}_{t-1})\\ h_{t}=w_{t}{\vec{h}}_{t}+\nu_{t}{\overset{\leftarrow }{h}}_{t}+b \end{array}\right.$$

### 3.2 PDO module

The hyperparameters of the BiGRU network (learning rate, batch size, number of neurons per layer, and number of layers) significantly affect its performance in data recovery. Selecting appropriate hyperparameters is crucial for enhancing the model’s training effectiveness and overall predictive accuracy. In view of this, this study employs the PDO module to optimize the BiGRU’s hyperparameters. The PDO algorithm is inspired by two primary behaviors of prairie dogs: foraging and burrowing (Ezugwu et al., 2022; Sun et al., 2024e). During the foraging phase, prairie dogs search for new food sources within a certain area and communicate the location of food to other individuals. They also estimate the required burrowing effort based on the quality of the discovered food. In the burrowing phase, prairie dogs move according to the shared food location information and hide in burrows to evade predators. The algorithm divides the total number of iterations into four equal stages: the first two simulate foraging behavior, while the last two simulate burrowing behavior. This staged approach allows PDO to balance exploration and exploitation dynamically, thereby enhancing the effectiveness of hyperparameter optimization.

During the foraging phase, the algorithm further divides the total number of iterations evenly. When the iteration count satisfies *t* < *Max_iter*/4, individuals explore new food sources across the entire search space. The position update method for this phase is given by Equation 3.


(3) $$X_{i+1,j+1}=\text{GBes}t_{i,j}-\text{CBes}t_{i,j}\cdot \rho -CX_{i,j}\cdot \text{Levy}(n)$$

Where *GBest_i,j_* represents the global best position, *ρ* denotes the food source alert level, *CX_i,j_* refers to the random cumulative effect of all individuals, and *CBest_i,j_* indicates the current best position. The function *Levy*(*n*) follows the *Levy* distribution, which enhances the diversity of food source exploration and strengthens the algorithm’s global search capability.

The calculation formulas for *GBest_i,j_* and *CX_i,j_* are presented in Equations 4 and 5, respectively.


(4) $$\text{CBes}t_{i,j}=\text{GBes}t_{i,j}\cdot \Delta +\frac{X_{i,j}\cdot \text{mean}(X_{n,m})}{\text{GBes}t_{i,j}\cdot ({\mathrm{ub}}_{j}-{\mathrm{lb}}_{j})+\Delta }$$

(5) $$CX_{i,j}=(\text{GBes}t_{i,j}-rX_{i,j})/(\text{GBes}t_{i,j}+\Delta )$$

Where *Δ* represents the difference between individuals; *ub* and *lb* denote the upper and lower bounds of the search space, respectively; *rX* refers to the position of a randomly selected individual.

When the iteration count satisfies *Max_iter*/4 ≤ t ≤ *Max_iter*/ 2, the algorithm enters the phase of evaluating food quality and determining the mining intensity, as detailed in Equation 6.


(6) $$X_{i+1,j+1}=\text{GBes}t_{i,j}\cdot \mathrm{rX}\cdot \mathrm{DS}\cdot \text{Levy}(n)$$

Where DS represents the mining intensity.

During the first half of the burrowing activities, when the iteration count satisfies *Max_iter* / 2 ≤ t < 3 *Max_iter* / 4, the algorithm evaluates the quality of the food sources. The position update method is as follows:


(7) $$X_{i+1,j+1}=\text{GBes}t_{i,j}-\text{CBes}t_{i,j}\cdot \varepsilon -CX_{i,j}\cdot \mathrm{rand}$$

Where *ε* represents the quality of the food source, and *rand* is a random number between 0 and 1.

When the iteration count satisfies 3*Max_iter* /4 ≤ t < *Max_iter*, the prairie dogs retreat to their burrows to observe predators.


(8) $$X_{i+1,j+1}=\text{GBes}t_{i,j}\times \mathrm{PE}\cdot \mathrm{rand}$$

Where *PE* represents the predator effect, as defined in Equation 9.


(9) $$\mathrm{PE}=1.5\times {\left(1-\frac{t}{\max_{-}\text{iter}}\right)}^{\left(\frac{2t}{\max_{-}\text{iter}}\right)}$$

**Figure 2:**
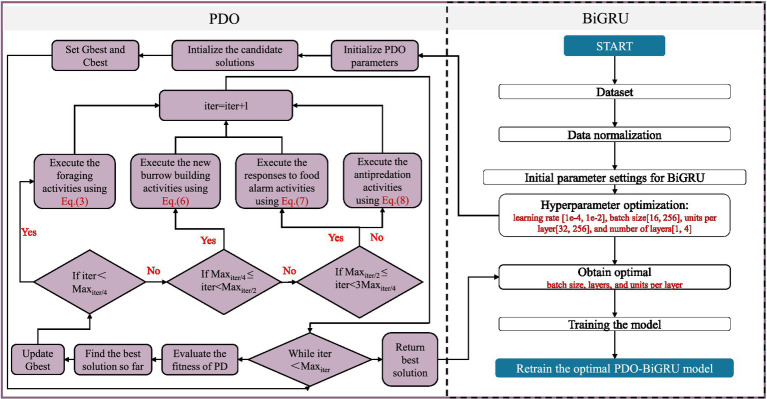
Flowchart of the PDO-BiGRU model.

Based on the above principle, this study integrates the PDO module with the BiGRU model. The PDO module systematically explores near-optimal combinations of hyperparameters (learning rate, batch size, the number of neurons per layer, and the number of network layers) by employing a predefined behavioral mechanism. Specifically, the learning rate is optimized within the range of [1e-4, 1e-2], ensuring a stable and efficient training process while preventing gradient explosion or slow convergence. The batch size is set between 16 and 256, enabling adaptive adjustments during mini-batch gradient descent; this facilitates efficient GPU memory utilization while preserving the model’s generalization capability. The number of layers is restricted to 1–4 to effectively mitigate gradient explosion during deep network training (Yang et al., 2016). The number of neurons per layer is optimized within the range of 32–256 to enhance the model’s capacity to capture temporal features while minimizing the risk of overfitting (Siami-Namini et al., 2019). In each iteration, the algorithm identifies a relatively optimal solution and updates the current configuration according to a specific replacement strategy. Through continuous iteration, the quality of the hyperparameter configuration progressively improves. Figure 2 illustrates the overall architecture of the PDO-BiGRU model.

### 3.3 GAN module

GANs are innovative deep learning architectures that demonstrate superior performance in data generation and complex distribution modeling tasks. The model consists of two main components: a generator and a discriminator. The generator learns the distribution characteristics of real data to produce highly similar synthetic samples, while the discriminator aims to distinguish whether the input data originates from the real dataset. These components are trained jointly through an adversarial process, where continuous competition drives ongoing improvements in model performance (Alqahtani et al., 2021). During training, the generator attempts to create samples that can “fool” the discriminator, whereas the discriminator strives to accurately differentiate between real and generated data. The loss functions for both components, which measure adversarial effectiveness and training convergence, are presented in Equations 10, 11.


(10) $$L_{G}=-E_{z\sim P_{z}}\{\log(D\cdot [G(z)])\}$$

(11) $$L_{D}=-E_{x\sim P_{r(x)}}\{\log[D(x)]\}-E_{z\sim P_{z}}\{\log\{1-D[G(z)]\}\}$$

Where *L_D_* and *L_G_* denote the discriminator and generator loss functions, respectively*_._ P_r_* is the true data distribution derived from the training set. *P_z_* is the distribution of the data generated by the generator. The variable *z* is a latent variable drawn from a predefined prior distribution. *D*(*x*) indicates the discriminator’s output score for a real data sample *x*. *G(z)* refers to the synthetic data sample produced by the generator given the input *z*. *D(G(z))* reflects the discriminator’s assessment of the generated data sample.

### 3.4 PDO-BiGRU-GAN network

This study proposes a time series imputation model—PDO-BiGRU-GAN—that integrates the PDO, BiGRU, and GAN. The model leverages BiGRU’s capability in capturing bidirectional temporal dependencies, PDO’s efficiency in hyperparameter optimization, and GAN’s potential in generating high-quality data. When addressing missing values in time series, the proposed approach demonstrates superior accuracy and robustness. In the modeling process, the generator receives time series data with missing values and utilizes a PDO-BiGRU architecture to extract bidirectional temporal features. Through adversarial training, it continuously generates data samples that increasingly resemble the real ones. Meanwhile, the discriminator distinguishes between generated and original samples, providing gradient feedback to the generator to enhance output quality. The training process is grounded in game-theoretic principles, where the generator and discriminator undergo iterative adversarial optimization, progressively enhancing the model’s imputation capability. To improve training stability, gradient penalty is applied to the discriminator. These strategies collectively reduce training oscillations and prevent overfitting, ensuring robust and reliable model convergence. The corresponding loss functions are defined in Equations 12–16.


(12) $$L_{R}=∥x⊙m-G(z)⊙m∥2$$

(13) $$L_{G2}=-D(\tilde{x})$$

(14) $$L_{C}=\text{Deviation}({\tilde{c}}_{t}^{f},{\tilde{c}}_{t}^{b})$$

(15) $$L_{G}=k\cdot L_{R}+L_{G2}+L_{C}$$

(16) $$L_{D}=-D(x)+D(\tilde{x})$$

Where *L_R_* denotes the reconstruction loss, which measures the discrepancy in alignment of the model-generated sequence with the original incomplete input. *L_C_* refers to the calibration loss, which quantifies the deviation between the forward- and backward-generated sequences in a time series. *L_G2_* represents the discriminator loss, which evaluates the authenticity of the generated data. *k* serves to modulate the relative impact of the discriminator loss compared to the reconstruction loss in the total loss formulation.

**Figure 3:**
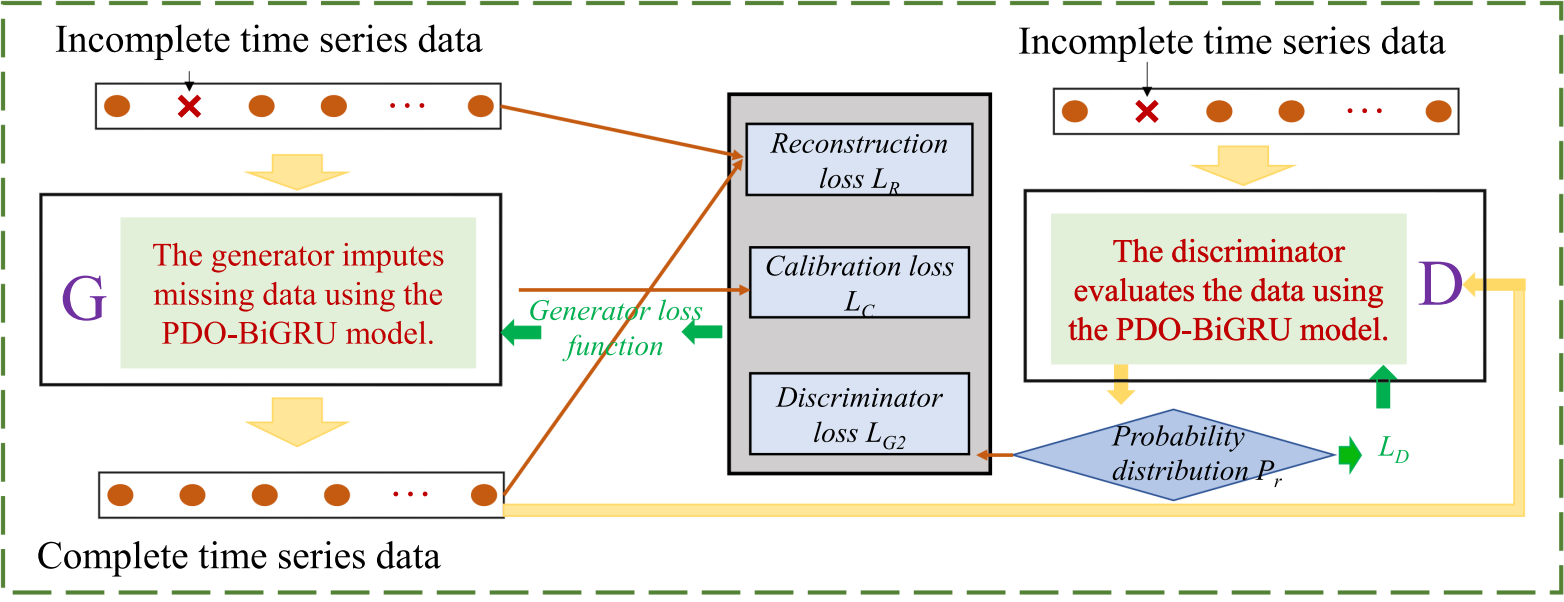
Illustration of the PDO-BiGRU-GAN network architecture.

Based on this approach, the study employs the PDO-BiGRU-GAN network to learn spatiotemporal dependencies from available data to infer missing values, thereby enabling the recovery of pipeline monitoring data. Figure 3 presents the network architecture, illustrating the workflow of the proposed model. The pseudocode of the PDO-BiGRU-GAN network is provided in Appendix 1.

## 4 Introduction to the engineering case

The data used in this study were obtained from an open-access monitoring database released by the project owner. All data were standardized according to a unified format to facilitate subsequent data analysis and model development. The implementation of the open-access mechanism has significantly improved the accessibility and reusability of engineering monitoring data, providing a reliable and authentic foundation for this research. A brief overview of the project background is presented below.

**Figure 4:**
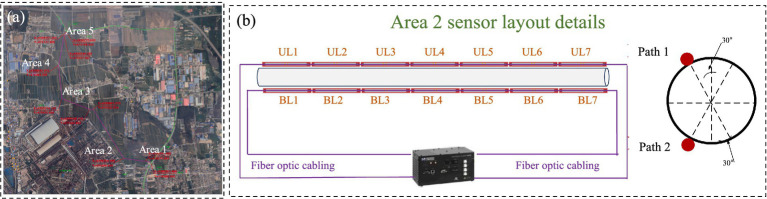
Pipeline layout and monitoring areas: **(a)** Five key areas; **(b)** Schematic diagram of sensor deployment in area.

This study is based on a typical open-access data project from a natural gas pipeline engineering initiative in Hebei Province, China. The project, organized and implemented by the owner, deployed an advanced fiber optic sensing monitoring system along an operational gas pipeline. This system covers five key monitoring areas to enable real-time and continuous surveillance of the pipeline’s operational status (Figure 4). Upon project completion, the collected monitoring data were made available to research institutions, providing a multidimensional platform for academic analysis and methodological validation.

This study focuses on Area 2 as the primary research area, emphasizing the analysis of monitoring data collected by long-gauge fiber Bragg grating (FBG) sensors within this area. A dual-end data acquisition mode is employed, which enhances system stability and improves fault tolerance in the event of single-point sensor failure. Figure 4B illustrates the layout of the long-gauge FBG sensors in Area 2. All sensors were installed in strict accordance with national technical standards and industry regulations (DB32/T 2880-2016, 2016; TSG D7005-2018, 2018). Data acquisition was performed using the MOI Sm125-500 demodulator, ensuring high precision and reliability. The construction cost of the monitoring system approached one million yuan. The system was completed and put into operation on November 1, 2023, with data collection commencing on the same day.

## 5 Database construction and preprocessing

### 5.1 Database construction

**Figure 5:**
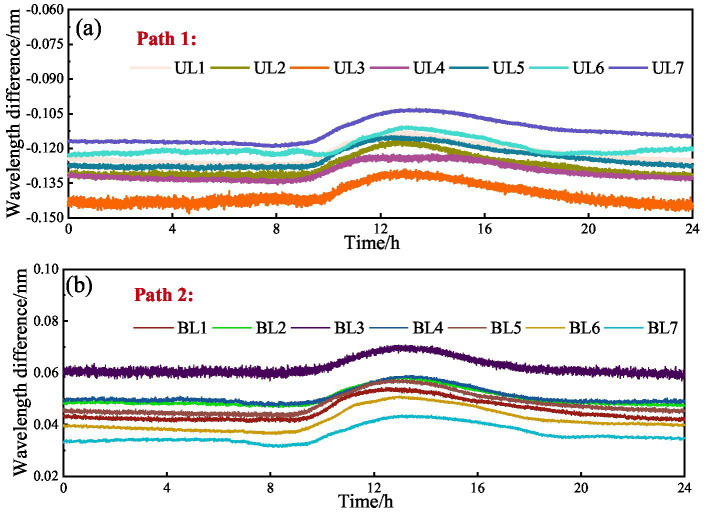
Database construction: **(a)** Path 1; **(b)** Path 2.

Section 3 briefly introduces the basic overview of the pipeline monitoring project. Based on the project’s open-source data, this study conducted relevant analyses. Fiber-optic monitoring data exhibit high sensitivity to environmental factors. Under normal weather conditions, the data primarily correlate with variations in temperature and pipeline deformation. However, under rainy conditions, the changes in fiber-optic monitoring data become more complex, influenced by multiple factors such as rainfall, temperature, pipeline deformation, and groundwater levels. As an initial exploration into pipeline monitoring data recovery, this study focuses on analyzing the feasibility of recovering fiber-optic monitoring data under normal weather conditions. Specifically, the data were collected on January 15, 2024, with sensors sampling at 1 Hz. A total of 86,400 data sets were obtained that day, each containing monitoring information from 14 sensors. Subsequently, a database was constructed based on this data set. Two methods were considered for database construction: (1) Strain-based method: This method derives strain data by removing the temperature component from wavelength shifts. However, it requires additional temperature sensors, which may introduce measurement errors and reduce the spatiotemporal consistency of the dataset. (2) Wavelength difference method: This method calculates the difference between the wavelength measured on the collection day and the reference wavelength recorded on November 1, 2023. Because wavelength shifts inherently reflect both strain and temperature effects, directly using wavelength differences better preserves the spatiotemporal characteristics of the sensor data and improves the reliability of data recovery. Given these advantages, this study adopts the wavelength difference method for database construction. The constructed database is shown in Figure 5.

On this basis, it is necessary to further examine the causes and potential types of missing data to enable the construction of an incomplete dataset. Unlike conventional electrical sensors, the FBG sensors used in this study include multiple measurement points along a single optical fiber. This configuration results in distinctive data loss mechanisms. The main causes are as follows: (1) construction activities near the pipeline may render multiple sensors within localized regions unavailable. (2) Optical fibers are typically spliced, and splice points are prone to breakage, leading to partial data loss. (3) Individual FBG sensors may fail due to damage or aging. In this case, the optical path remains intact, but valid measurements cannot be obtained (Wu et al., 2020). (4) Signal attenuation occurs during long-distance optical transmission. Without proper amplification, excessive attenuation prevents correct data interpretation at the receiving end, resulting in data loss (Torres et al., 2011). Collectively, these factors contribute to the occurrence of missing data in pipeline monitoring.

**Figure 6:**
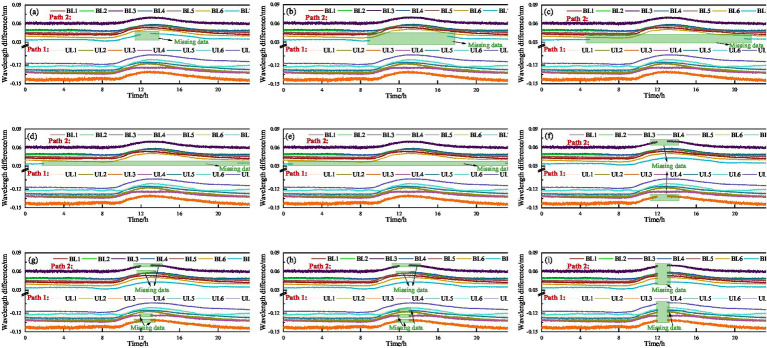
Illustration of the incomplete dataset: **(a)** Missing data ratio 1/24; **(b)** Missing data ratio 8/24; **(c)** Missing data ratio 16/24; **(d)** Missing data ratio 20/24; **(e)** Missing data ratio 22/24; **(f)** Missing data from 2 sensors; **(g)** Missing data from 4 sensors; **(h)** Missing data from 8 sensors; **(i)** Missing data from 14 sensors.

FBG monitoring systems typically perform signal demodulation via single-end wiring. However, a break in the optical fiber may prevent downstream sensors from functioning. To mitigate this risk, a dual-end redundant wiring strategy was implemented in this study (Figure 4). This strategy ensures that a fault in one sensor does not compromise the monitoring of others, thereby minimizing the overall system impact. Based on this sensor architecture and relevant literature (Jiang et al., 2022; Tien et al., 2024), missing sensor data are classified into two types: single-sensor loss and multiple-sensor loss. To investigate these scenarios, several incomplete datasets were constructed to simulate different conditions: (1) Single-sensor loss with proportions of 1/24, 8/24, 16/24, 20/24, and 22/24; and (2) Multiple-sensor loss involving 2/14, 4/14, 8/14, and 14/14 sensors. Figure 6 illustrates the incomplete datasets, while Table 1 summarizes key information. This completes the construction of the incomplete database.

**Table 1:** Statistics of the incomplete dataset.

Statistical information		Corresponding results
Sampling frequency		1HZ
Total number of sensors		14个
Types of missing data	Different missing data ratios	1/24
		8/24
		16/24
		20/24
		22/24
	Different numbers of missing sensors	2 (BL3, UL3)
		4 (BL3, UL3, BL4, UL4)
		8 (BL2, UL2, BL3, UL3, BL4, UL4, BL5, UL5)
		14

### 5.2 Database preprocessing

Section 5.1 presents the construction of various types of incomplete datasets based on engineering data. Before inputting these multi-condition incomplete datasets into the PDO-BiGRU-GAN network, data normalization is a critical preprocessing step (Liu et al., 2025b; Sun et al., 2025d). Normalization eliminates dimensional inconsistencies among features, ensuring that variables vary within comparable numerical ranges (Lv et al., 2023; Sun et al., 2023b). This reduces the risk of gradient shift and enhances model stability during training. Moreover, mapping raw data to a unified scale improves training efficiency, accelerates convergence, and mitigates the likelihood of the model becoming trapped in local minima (Li et al., 2025b). The specific normalization formula is provided in Equation 17 (Xie et al., 2025a).


(17) $$G_{i}=\frac{Q_{i}-Q_{\min}}{Q_{\max}-Q_{\min}}$$

Where *Q*_i_ and *G_i_* are the original and normalized values of measured data, respectively. *Q*_max_ and *Q*_min_ are the maximum and minimum values of measured data, respectively.

## 6 Analysis of data recovery results based on the PDO-BiGRU-GAN network

Using incomplete datasets and the PDO-BiGRU-GAN network, this study investigates data recovery performance under various missing data scenarios. The experiments were conducted on the TensorFlow platform with hardware comprising 256 GB of memory, an NVIDIA TITAN X (Pascal) GPU, and two Intel Xeon(R) E5-2696 v4 processors, ensuring efficient handling of large-scale computations. Specifically, Section 6.1 analyzes the model’s hyperparameter sensitivity to demonstrate the necessity of integrating the PDO module. Section 6.2 employs ablation experiments to evaluate the contribution of each module to overall performance. Section 6.3 examines recovery performance across different missing data ratios (1/24, 8/24, 16/24, 20/24, and 22/24). Section 6.4 further assesses recovery under multiple sensor missing scenarios (2/14, 4/14, 8/14, and 14/14). Section 6.5 examines the model’s computational time. Additionally, to comprehensively evaluate the proposed model’s effectiveness, it is compared against eight existing deep learning methods.

### 6.1 Hyperparameter sensitivity analysis

This study employs the PDO module to optimize four key hyperparameters: learning rate, batch size, units per layer, and number of layers. The rationale for focusing on these hyperparameters is explained as follows. The learning rate controls the speed of parameter updates. An excessively high learning rate can cause oscillation or divergence, while a rate that is too low may result in slow convergence or entrapment in local optima (Dohare et al., 2024). Batch size directly affects both generalization and computational efficiency. Smaller batches increase the stochasticity of gradient estimates, thereby enhancing generalization, whereas larger batches enable faster computation and more stable convergence (Offiong et al., 2023). The number of neurons per layer determines the representational capacity of each layer. Too few neurons can lead to underfitting, while too many may cause overfitting and substantially increase computational cost. Network depth, indicated by the number of layers, reflects the model’s capacity for feature extraction. Shallow networks may fail to capture long-term dependencies, while excessively deep networks can suffer from vanishing gradients, overfitting, and training instability (Offiong et al., 2023). Given these considerations, the PDO module focuses on optimizing these four hyperparameters to enhance model performance while controlling computational costs. In contrast, secondary hyperparameters, such as dropout rate or regularization coefficients, exert only indirect effects. Including them would significantly expand the search space, potentially increasing computational costs and reducing optimization efficiency (Bian and Priyadarshi, 2024). Therefore, this study excludes them from the optimization process.

**Figure 7:**
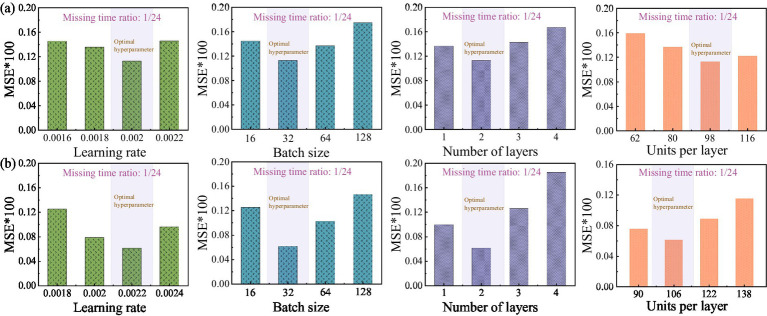
Hyperparameter sensitivity analysis: **(a)** Missing data ratio 1/24; **(b)** 2 Missing sensors.

To evaluate the effectiveness of the PDO module in hyperparameter optimization, this study conducts a hyperparameter sensitivity analysis. This approach assesses the model’s performance variations across different hyperparameter configurations, thereby demonstrating the necessity of integrating the PDO module. Table 2 presents the configurations of learning rate, batch size, units per layer, and number of layers automatically selected by the PDO module across various recovery tasks. These results indicate that the PDO module can select appropriate hyperparameter combinations based on task characteristics. Specifically, when the missing ratio is relatively low (e.g., 1/24), the model tends to adopt a higher learning rate (0.02), a smaller batch size (32), a shallow two-layer structure, and an asymmetric distribution of units (98). This “low-capacity–shallow” configuration reduces complexity and mitigates overfitting while preserving temporal features. In contrast, under a higher missing ratio (e.g., 20/24), the model prefers a lower learning rate (0.0011), a larger batch size (128), a larger number of units (196), and a deeper three-layer stacked structure. Such a configuration enhances the generative adversarial network’s ability to model sparse data and enables it to capture long-term dependencies through increased depth. Further analysis involves varying each hyperparameter sequentially to assess the tuning effect of the PDO module, as shown in Figure 7. It is evident that the hyperparameter configurations selected by the PDO module yield the lowest MSE, thereby achieving optimal tuning and enhanced model performance. Overall, the hyperparameter sensitivity analysis demonstrates that the PDO module exhibits strong adaptability to varying task complexities by dynamically adjusting hyperparameter settings, thereby enhancing the accuracy of data recovery.

**Table 2:** Hyperparameter optimization results.

Hyperparameters	Missing data ratio					Multiple-sensor data loss			
	1/24	8/24	16/24	20/24	24/24	2 Missing sensors	4 Missing sensors	8 Missing sensors	14 Missing sensors
Learning rate	0.002	0.0018	0.0016	0.0013	0.0011	0.0022	0.0018	0.0018	0.0012
Batch size	32	64	64	64	128	32	64	64	128
Units per layer	98	122	148	175	196	106	130	164	208
Number of layers	2	3	3	3	3	2	2	3	3

### 6.2 Ablation study analysis

**Figure 8:**
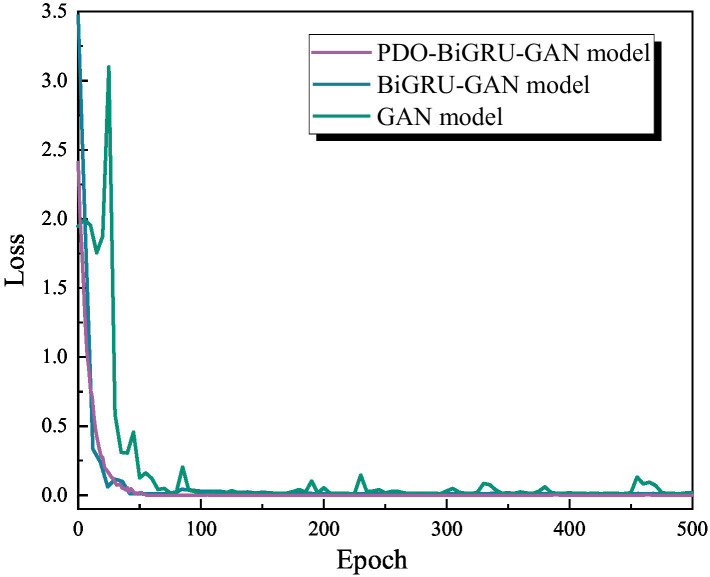
Loss curves of the three models (GAN, BiGRU-GAN, and PDO-BiGRU-GAN) under a missing data ratio of 1/24.

Ablation experiments selectively remove specific components of a model to evaluate their impact on overall performance. This approach effectively confirms the necessity and contribution of each module in the model architecture. Based on this methodology, the present study analyzed three models—GAN, BiGRU-GAN, and PDO-BiGRU-GAN—via selective module removal (Table 3). Initially, loss curves were plotted for the three models under a missing data ratio of 1/24 (Figure 8). The basic GAN model exhibited a rapid decrease in loss; however, its loss curves displayed pronounced fluctuations, indicating instability in missing data recovery tasks. Incorporating the BiGRU module (BiGRU-GAN) produced smoother loss curves, demonstrating that the integration of temporal information enhances model stability and generalization. Further addition of the PDO module in PDO-BiGRU-GAN achieved more favorable convergence characteristics. Loss decreased rapidly and stabilized at a low level, indicating that PDO-based hyperparameter optimization significantly improves training efficiency and overall model performance. The contributions of each component were further assessed under varying data missing ratios of 1/24, 8/24, and 16/24 (Table 3). The results indicate that removing either the PDO module or the BiGRU structure degrades the model’s predictive capability. Specifically, when the missing data ratio ranges from 1/24 to 16/24, excluding the PDO module leads to increases in mean absolute error (MAE) by 9.80–21.88%, root mean square error (RMSE) by 18.30–23.05%, mean absolute percentage error (MAPE) by 16.72–27.23%, and MSE by 33.26–40.79%, while coefficient of determination (R^2^) decreases by 0.53–1.00%. Further removal of the BiGRU structure causes more pronounced performance deterioration: MAE increases by 53.67–60.03%, RMSE by 42.28–61.73%, MAPE by 42.35–57.07%, MSE by 66.68–85.35%, and R^2^ declines by 3.27–5.08%. These findings indicate that the GAN module provides fundamental generative capability, the BiGRU structure captures temporal dependencies to improve reconstruction accuracy, and the PDO module optimizes key hyperparameters to enhance training efficiency and generalization (Ezugwu et al., 2022). The synergistic effect of these three components enables PDO-BiGRU-GAN to achieve optimal performance in data imputation tasks, confirming the necessity and contribution of each module in the model architecture.

**Table 3:** Ablation study results.

Model	Module			Proportion	Performance metrics				
	GAN	KOA	GPS		MAPE	MSE*100	MAE*10	RMSE*10	R^2^
GAN (baseline)				1/24	0.10232	0.33849	0.45093	0.5818	0.9588
	**√**			8/24	0.11543	0.7928	0.86601	0.89039	0.9421
				16/24	0.19215	1.25729	1.01144	1.12129	0.94
BiGRU-GAN				1/24	0.07082	0.19048	0.23163	0.43643	0.985
	**√**	**√**		8/24	0.08376	0.18989	0.44451	0.43577	0.98
				16/24	0.10064	0.33225	0.51753	0.57641	0.9778
PDO-BiGRU-GAN				1/24	0.05898	0.11278	0.20892	0.33582	0.9902
	**√**	**√**	**√**	8/24	0.06095	0.11613	0.37233	0.34078	0.98994
				16/24	0.08249	0.22174	0.40428	0.4709	0.9872

### 6.3 Data recovery results of the PDO-BiGRU-GAN model under different missing data ratios

**Figure 9:**
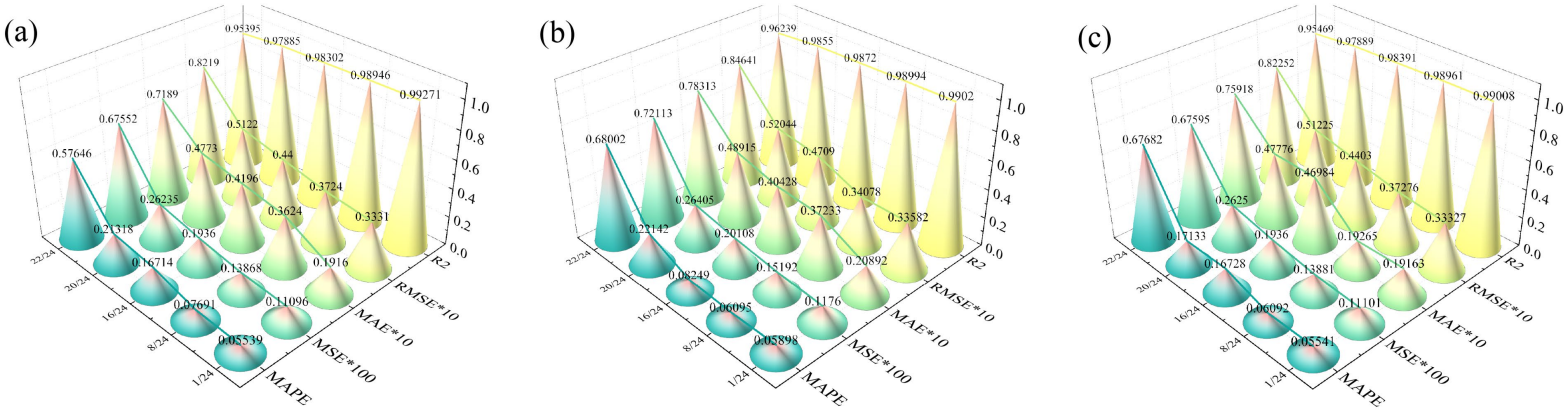
Data recovery results based on the PDO-BiGRU-GAN network: **(a)** BL1; **(b)** BL4; **(c)** BL7.

The pipeline project employed a total of 14 sensors. This section focuses on analyzing three sensors: BL1, BL4, and BL7. The recovery performance of these sensors was evaluated using the PDO-BiGRU-GAN model under varying missing data ratios of 1/24, 8/24, 16/24, 20/24, and 22/24. Figure 9 illustrates five performance metrics of data recovery based on the PDO-BiGRU-GAN model. Notably, all data were normalized to eliminate the influence of differing measurement units (Liu et al., 2025c; Sun et al., 2025e). Overall, the PDO-BiGRU-GAN model demonstrated strong recovery capabilities across all missing data ratios, with the R^2^ consistently above 0.95. Further analysis revealed that as the missing data ratio increased, error metrics (MSE, RMSE, MAPE, and MAE) showed an increasing trend, while R^2^ values gradually decreased. Particularly, a sharp decline in model performance occurred when the missing ratio increased from 20/24 to 22/24. This performance drop is attributed to the significant reduction of available historical data, which impairs the model’s ability to capture the intrinsic temporal patterns of the time series, thus weakening the imputation effect. Under conditions of extreme data sparsity, the model struggles to accurately restore complex features, resulting in substantially higher error metrics and notably lower R^2^ values. Therefore, when employing the PDO-BiGRU-GAN model for data recovery, the missing data ratio within incomplete data windows should be maintained below 20/24 to ensure optimal recovery performance.

This section presents a detailed comparison between the proposed PDO-BiGRU-GAN model and eight representative data recovery models, aiming to demonstrate its superior performance. These models span from basic generative frameworks to advanced architectures, providing a comprehensive overview of mainstream techniques in time-series recovery tasks. The selected models include the traditional GAN, GRU-GAN, LSTM-GAN, CNN-GRU-GAN, CNN-LSTM-GAN, Bi-GRU-GAN, Bi-LSTM-GAN, and STOA-Bi-LSTM-GAN. These models span from basic generative frameworks to advanced architectures, providing a comprehensive overview of commonly used techniques in time-series recovery tasks. Specifically, the traditional GAN serves as a baseline generative model. GRU-GAN and LSTM-GAN emphasize unidirectional temporal dependencies, making them suitable for capturing long-term trends. Bi-GRU-GAN and Bi-LSTM-GAN capture both past and future dependencies through bidirectional sequence modeling, thereby improving recovery accuracy for rare events or edge cases. CNN-GRU-GAN and CNN-LSTM-GAN extract local temporal features via convolutional layers and combine them with recurrent structures to model multivariate dependencies, thereby enhancing recovery of complex patterns. The STOA-Bi-LSTM-GAN represents the state-of-the-art approach, integrating bidirectional recurrent modeling with optimized training strategies to achieve greater adaptability and stability. To ensure a fair comparison between PDO-BiGRU-GAN and the eight baseline models, all models were trained under identical data preprocessing and normalization conditions (Sun et al., 2024f; Xie et al., 2025b), and with the same computational resources. Notably, PDO-BiGRU-GAN employs the PDO algorithm for automated optimization, whereas STOA-Bi-LSTM-GAN uses the STOA algorithm. The hyperparameters of the remaining seven baseline models were not taken directly from literature values, as optimal settings can vary across different data recovery tasks. Instead, a grid search was conducted to tune these models, ensuring they achieved their best possible performance for the current task and maintaining the objectivity of the comparison.

**Figure 10:**
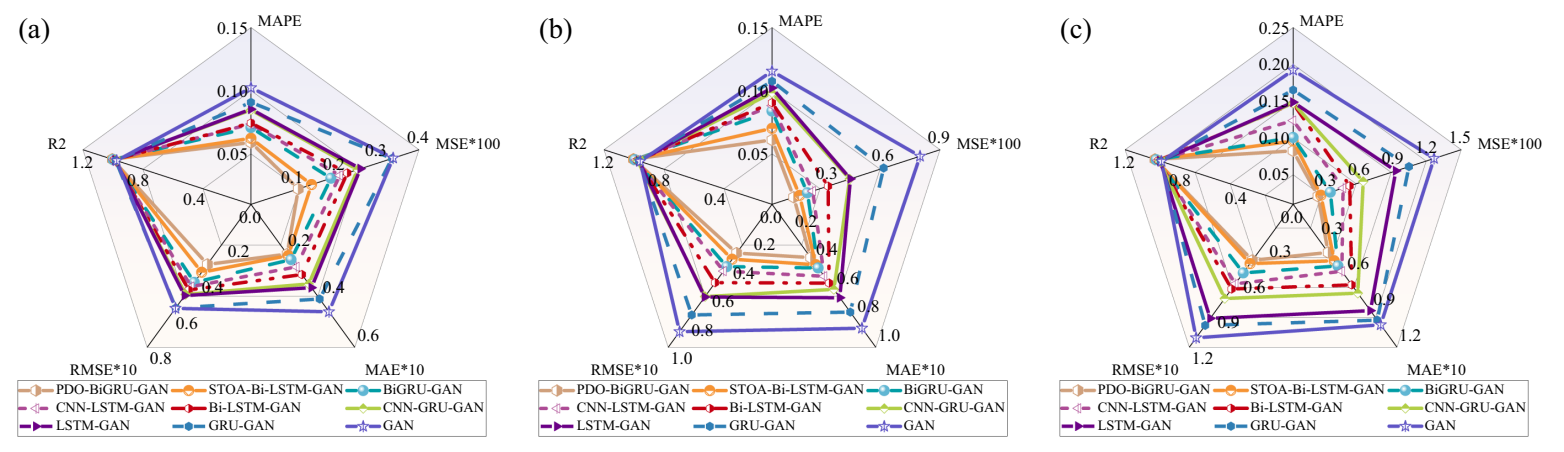
Performance comparison between PDO-BiGRU-GAN and eight models: **(a)** 1/24; **(b)** 8/24; **(c)** 16/24.

For clarity, this study selected the recovery results of sensor BL4 under three missing data ratios: 1/24, 8/24, and 16/24. Figure 10 presents radar charts of the performance metrics for all models under these conditions. The results indicate that PDO-BiGRU-GAN consistently achieves the lowest error metrics (MSE, RMSE, MAPE, MAE) and the highest R^2^ across all three scenarios, demonstrating its superior data recovery capability. In contrast, the remaining eight models exhibit varying degrees of performance degradation across the five evaluated metrics. For example, at a missing ratio of 1/24, the PDO-BiGRU-GAN model attained an R^2^ of 0.9902, MSE of 0.001128, RMSE of 0.03358, MAPE of 0.05898, and MAE of 0.02089. Compared to other models, PDO-BiGRU-GAN improved R^2^ by 0.197–3.28%, reduced MSE by 21.77–66.68%, RMSE by 11.55–42.28%, MAPE by 5.25–42.36%, and MAE by 2.93–53.67%. To verify the statistical significance of these performance differences, Wilcoxon signed-rank tests were conducted on R^2^, MAE, RMSE, MSE, and MAPE [see Taheri and Hesamian (2013) and Woolson (2007) for calculation details]. The results indicate that PDO-BiGRU-GAN significantly outperforms all eight baseline models across all metrics (*p* < 0.05), confirming that its performance improvements are statistically robust and not due to random variation. Overall, the PDO-BiGRU-GAN model exhibits optimal recovery performance across various missing data conditions.

### 6.4 Data recovery results of the PDO-BiGRU-GAN model under different numbers of missing sensors

**Figure 11:**
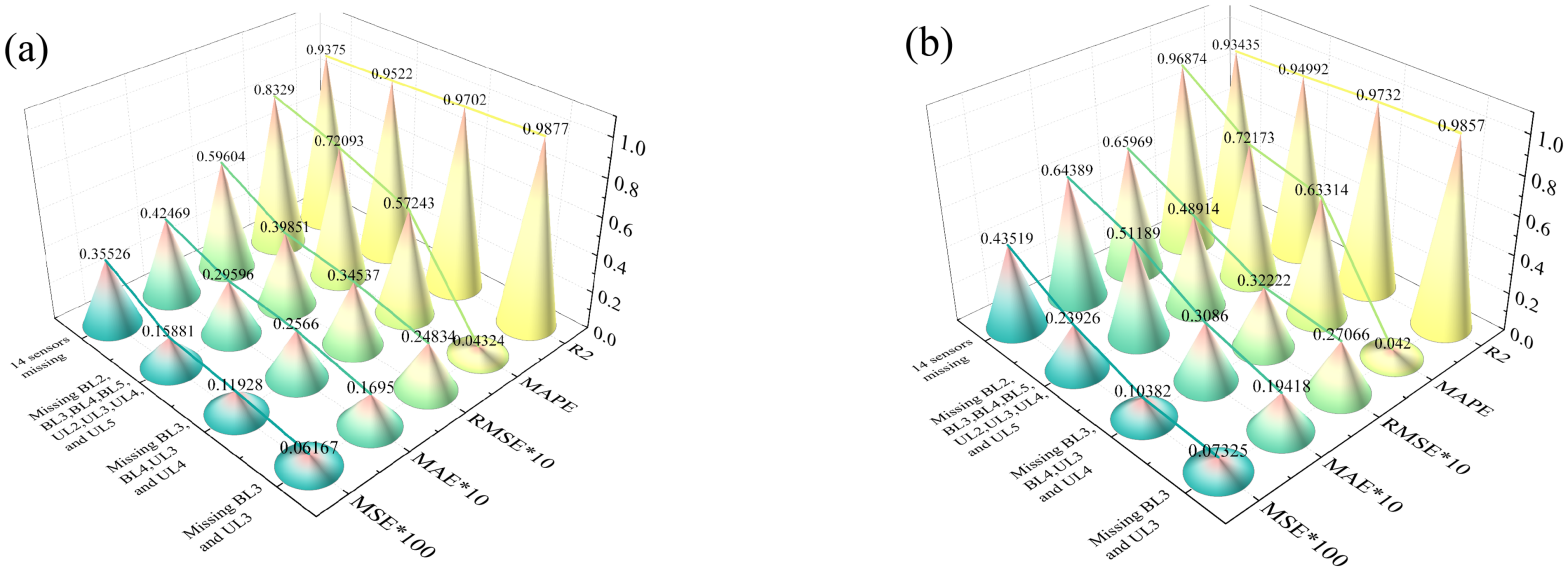
Data recovery results for sensors UL3 and BL3 with multiple missing sensors using the PDO-BiGRU-GAN model: **(a)** UL3; **(b)** BL3.

To assess the model’s robustness in the face of multi-sensor data loss, this section investigates the recovery capabilities of the PDO-BiGRU-GAN model under varying degrees of sensor unavailability. Four missing data scenarios were designed: missing 2 sensors (BL3, UL3), missing 4 sensors (BL3, UL3, BL4, UL4), missing 8 sensors (BL2, UL2, BL3, UL3, BL4, UL4, BL5, UL5), and missing 14 sensors. In all scenarios, data from UL3 and BL3 were reconstructed. To simplify the analysis, only the recovery results of sensors UL3 and BL3 were evaluated. Figure 11 presents the performance metrics of the PDO-BiGRU-GAN model for recovering UL3 and BL3 under different levels of sensor loss. All performance metrics were computed using normalized values to ensure comparability and mitigate the impact of differences in data magnitude. Overall, the results indicate that the PDO-BiGRU-GAN model consistently demonstrates strong recovery performance across all scenarios. The R^2^ values remain generally above 0.93, suggesting that the model effectively captures spatiotemporal features and reconstructs missing data by leveraging latent dynamic correlations among sensors. However, as the number of missing sensors increases, the R^2^ value gradually decreases. Meanwhile, error metrics (MSE, RMSE, MAPE, MAE) increase accordingly, indicating a decline in model performance. This degradation can be attributed to two primary factors: the diminishing volume of available reference data and the growing complexity of underlying data patterns, both of which increase the difficulty of accurately estimating missing values. Despite the observed degradation as the number of missing sensors increases, the PDO-BiGRU-GAN model still exhibits remarkable recovery performance when dealing with multi-sensor data loss. Its advanced capacity for learning temporal–spatial dependencies and modeling nonlinear relationships makes it particularly suitable for recovering critical sensor information under complex operating conditions. These advantages highlight the model’s significant potential for application in intelligent monitoring systems and structural health diagnostics, offering both wide applicability and notable engineering value.

**Figure 12:**
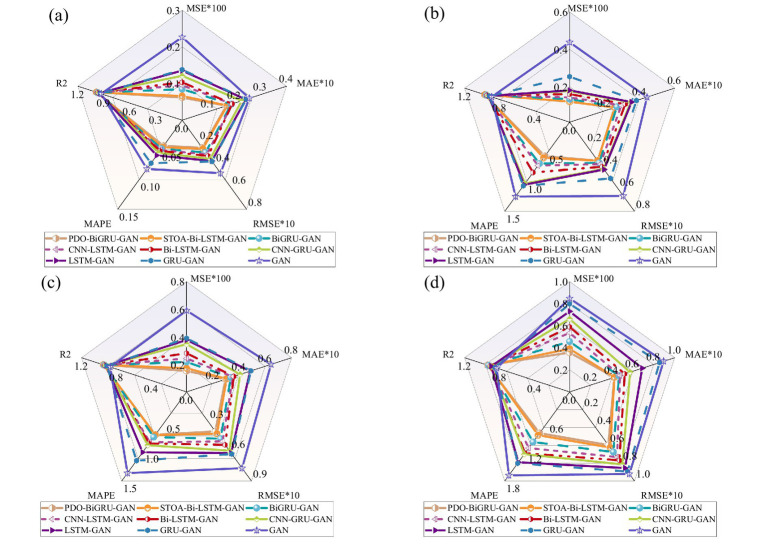
Data recovery performance of UL3 under multiple missing sensors: **(a)** 2 Missing sensors; **(b)** 4 Missing sensors; **(c)** 8 Missing sensors; **(d)** 14 Missing sensors.

Furthermore, this section provides a comprehensive evaluation of the PDO-BiGRU-GAN model’s recovery performance compared to eight other models under multi-sensor data loss conditions. For brevity, sensor UL3 is chosen as the representative for multi-model comparison. Figure 12 presents the performance metrics of PDO-BiGRU-GAN and the eight compared models. The results demonstrate that PDO-BiGRU-GAN consistently achieves the best performance across all evaluation metrics, exhibiting the lowest error levels and the highest R^2^ values. For example, with eight sensors missing, PDO-BiGRU-GAN attains an R^2^ of 0.9522, MSE of 0.001588, RMSE of 0.03985, MAPE of 0.7209, and MAE of 0.02960. Compared to other models, its R^2^ improves by 0.197 to 3.28%; MSE decreases by 21.77 to 66.68%; RMSE reduces by 11.55 to 42.28%; MAPE declines by 5.25 to 42.36%; and MAE lowers by 2.93 to 53.67%. Consistent with Section 6.3, Wilcoxon signed-rank tests were conducted on the five performance metrics across different numbers of missing sensors. The results show that PDO-BiGRU-GAN outperforms all eight comparison models, with *p*-values below 0.05 for all metrics, indicating that the performance differences are statistically significant. Overall, the comparison among the nine models indicates that PDO-BiGRU-GAN maintains superior recovery accuracy for UL3 data across different sensor loss scenarios. Furthermore, the model shows similar stable advantages in recovering data from other sensors; however, these results are not detailed here due to space constraints. In summary, PDO-BiGRU-GAN demonstrates excellent imputation capability under multi-sensor data loss conditions and represents a promising approach for this problem.

### 6.5 Computational efficiency analysis

**Figure 13:**
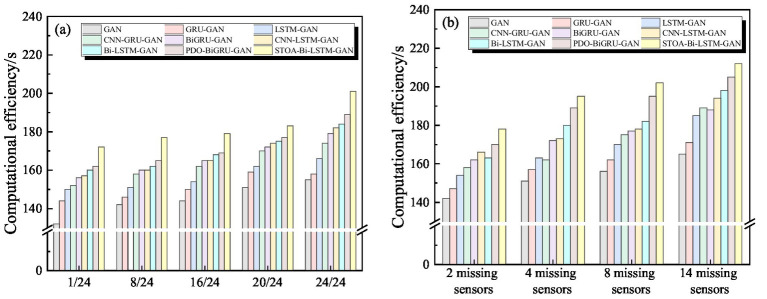
Computation time comparison of nine models across different data recovery tasks: **(a)** Varying missing data ratios; **(b)** Multiple missing sensors.

The PDO-BiGRU-GAN model has a relatively complex architecture and many parameters, resulting in higher computational costs during training and inference. To evaluate its feasibility and deployment potential in practical applications, a systematic assessment of its computation time is necessary. This section compares the computation time of PDO-BiGRU-GAN with eight existing methods under identical task conditions, as shown in Figure 13. Compared with simpler models (standard GAN, GRU-GAN, and LSTM-GAN), PDO-BiGRU-GAN’s computation time increases by approximately 8.77 to 15.11%. Despite this increase, the additional cost is acceptable given the significant improvement in recovery accuracy. Further comparisons show that, relative to more complex architectures (CNN-GRU-GAN, CNN-LSTM-GAN, Bi-LSTM-GAN, and BiGRU-GAN), PDO-BiGRU-GAN’s computation time increases only by 1.25 to 7.32%, indicating that slight computational overhead yields substantial performance gains. Additionally, relative to the most complex STOA-Bi-LSTM-GAN model, PDO-BiGRU-GAN reduces computation time by approximately 3.17 to 7.27%. Overall, PDO-BiGRU-GAN incurs only a slight increase in computational cost compared to simpler models, while outperforming the most complex ones in efficiency. This advantage results from two main factors. First, the PDO algorithm employs a more efficient hyperparameter search strategy, significantly reducing ineffective computations during training (Biswas et al., 2024; Izci et al., 2024). Second, the model architecture preserves essential feature extraction capabilities while avoiding redundant layer stacking, effectively controlling resource consumption and ensuring improved recovery efficiency. In summary, PDO-BiGRU-GAN achieves substantial improvements in recovery accuracy while maintaining controlled computation time, making it a promising model for pipeline monitoring data recovery tasks.

### 6.6 Summary of comparative analysis between PDO-BiGRU-GAN and eight existing models

Sections 6.3–6.5 systematically compare the performance of PDO-BiGRU-GAN with eight existing models—GAN, GRU-GAN, LSTM-GAN, CNN-GRU-GAN, CNN-LSTM-GAN, BiGRU-GAN, BiLSTM-GAN, and STOA-BiLSTM-GAN—on data reconstruction tasks. This section further summarizes the advantages and limitations of each model and the trade-offs between accuracy and computational cost, as shown in Table 4. In the pipeline monitoring project examined in this study, PDO-BiGRU-GAN emerged as the optimal model based on a trade-off between accuracy and computational efficiency. For future research, investigators can select an appropriate model based on the characteristics in Table 4 and the specific requirements of their projects for data reconstruction.

**Table 4:** Comparative analysis of the proposed PDO-BiGRU-GAN model and eight existing models.

Model	Technical advantage	Limitations	Accuracy–computational cost trade-off
GAN	Basic generative modeling for missing data recovery	No temporal dependency modeling, limited sequence feature capture	Low accuracy, Very Low computational cost
GRU-GAN	Unidirectional GRU captures short-term temporal patterns	Limited ability for long-term dependencies	Moderate accuracy, Low computational cost
LSTM-GAN	Unidirectional LSTM captures long-term dependencies	Limited local feature extraction	Moderate-high accuracy, Moderate-Low computational cost
CNN-GRU-GAN	Convolution extracts local features + GRU handles temporal dependencies	Complex architecture, higher training cost	High accuracy, Moderate-High computational cost
CNN-LSTM-GAN	Convolution + LSTM captures both local and long-term dependencies	Large number of parameters, long training time	High accuracy, High computational cost
Bi-GRU-GAN	Bidirectional GRU captures past and future dependencies	Weak in local feature extraction	High accuracy, Moderate-High computational cost
Bi-LSTM-GAN	Bidirectional LSTM captures short- and long-term dependencies	Complex architecture, relatively long training time	High accuracy, High computational cost
STOA-Bi-LSTM-GAN	Bidirectional LSTM + STOA hyperparameter optimization	Hyperparameter search overhead, long training time	Very high accuracy, Very High computational cost
PDO-BiGRU-GAN	Bidirectional LSTM + STOA hyperparameter optimization	Computational cost during hyperparameter optimization	Very high accuracy, High computational cost; similar or better precision than STOA with lower overhead

## 7 Discussion

This study tackles the prevalent issue of missing data in pipeline monitoring by proposing a novel PDO-BiGRU-GAN framework. The framework integrates three key components: the PDO module for hyperparameter optimization, the BiGRU module for temporal feature extraction, and the GAN module for data generation and distribution approximation. To validate the method, a pipeline monitoring dataset was established using field data collected from actual pipeline projects. The study first analyzes the model’s sensitivity to hyperparameters, demonstrating the necessity of the PDO module in the optimization process. Ablation experiments were then conducted to assess the independent contribution of each module. Furthermore, the proposed model is compared with eight mainstream deep learning models in terms of prediction accuracy and computational efficiency. Overall, the PDO-BiGRU-GAN framework effectively reconstructs missing information in pipeline monitoring from a data-driven perspective, thereby providing more complete and reliable support for pipeline performance evaluation.

Although the proposed PDO-BiGRU-GAN model demonstrates high accuracy in recovering missing data in pipeline monitoring, it has several key limitations. These limitations can be categorized into three areas: the model itself, engineering applicability, and the intelligence of the pipeline monitoring system. Regarding the limitations of the model itself, the PDO-BiGRU-GAN model can generate samples that closely match the statistical characteristics of real data. However, it may struggle to capture rare fault patterns or extreme anomalies, such as those occurring under heavy rain, snow, or pipeline malfunctions. This limitation could potentially pose risks in pipeline monitoring. Future research could focus on modeling such exceptional operating conditions to enhance the model’s learning capability and robustness. Due to the focus and length constraints of the current study, the model’s performance across datasets of varying scales was not examined. Broader investigations into diverse data loss scenarios are required to address this gap. The approach also heavily depends on training data. When historical data are biased or incomplete, the model may produce misleading patterns, compromising the reliability of decisions. To mitigate this, future studies could incorporate physical model constraints, expert knowledge, or multi-source data into the PDO-BiGRU-GAN framework. This would reduce reliance on single historical datasets and improve both anomaly detection and overall predictive performance. Additionally, due to space limitations, this study only explored the combination of GAN and BiGRU modules and validated its effectiveness. Future research could explore the integration of GANs with other recurrent architectures, such as Transformer-based models or temporal convolutional networks, to assess data recovery performance across different frameworks. Finally, the PDO-BiGRU-GAN model is inherently a black-box model, lacking transparency and interpretability in its generation process. In practical applications, false positives or false negatives could complicate responsibility assignment and regulatory compliance. Future work could incorporate interpretability-enhancing techniques, coupled with uncertainty quantification and human-in-the-loop verification, to ensure the safety and reliability of data recovery.

Regarding engineering applicability, several limitations should be noted. First, the model has only been validated on the pipeline project in Tangshan, Hebei. Its generalizability across different pipelines or regions remains unassessed. Future studies could investigate the model’s transferability and adaptability across various pipeline types, geographic regions, and operating conditions. Second, the model requires significant computational resources during training, limiting direct deployment on low-power devices. In this study, it required approximately 2 GB of GPU memory and 4.9 GFLOPs. Future work could explore model lightweighting, parameter compression, and efficient inference strategies to enable deployment on low-power or edge devices. Third, the model can function as a data recovery module within pipeline monitoring systems and can be seamlessly integrated with existing optical fiber or other sensor data acquisition systems. While training in this study was conducted on a high-performance workstation, practical deployment can leverage a single GPU or high-performance CPU depending on data scale and real-time requirements, meeting computational demands for data recovery. From a software perspective, the model was developed on the TensorFlow platform and can interface with existing industrial pipeline monitoring systems, supporting deployment and extension across mainstream deep learning frameworks. Finally, this study applied the model solely to optical fiber sensor data recovery. Future research could extend the model to other sensor types, such as pressure, flow, and temperature, to systematically evaluate its applicability, stability, and cross-sensor generalization. This would further validate the model’s versatility across multi-source heterogeneous monitoring data.

In the context of intelligent pipeline monitoring, blockchain technology can be leveraged to enhance system reliability and automation. It ensures the integrity and traceability of both raw sensor data and AI-reconstructed data. Moreover, smart contracts can automatically trigger alerts or initiate maintenance actions based on recovered signals. For example, when a recovered signal indicates a potential risk, a smart contract can immediately activate warning mechanisms or execute pre-defined maintenance tasks, thereby reducing manual intervention and improving response efficiency. Furthermore, blockchain-based decentralized learning frameworks can improve privacy and robustness in multi-site deployments (Ressi et al., 2024b). Overall, the integration of blockchain, artificial intelligence, and other advanced technologies holds significant promise for advancing intelligent pipeline monitoring and providing a more robust technical foundation for the long-term safety of pipelines.

## 8 Conclusion

This study developed a novel PDO-BiGRU-GAN network to efficiently recover missing pipeline data. The model’s performance was evaluated using real engineering monitoring data under various types of data loss. The main findings are summarized as follows:

1. This study developed a novel PDO-BiGRU-GAN network, which integrates the hyperparameter optimization capability of the PDO module, the temporal feature extraction strength of the BiGRU module, and the data generation and imputation functionality of the GAN module. The proposed network was subsequently applied to recover missing data in pipeline monitoring systems.
2. Using an open-access pipeline project, a pipeline monitoring dataset was obtained. This dataset was employed to evaluate the proposed PDO-BiGRU-GAN network. Hyperparameter sensitivity analysis and ablation experiments were conducted to assess the model. The sensitivity analysis demonstrated that the PDO module substantially improved model performance by guiding optimal hyperparameter selection. Ablation experiments further showed that removing either the PDO or BiGRU module led to significant performance degradation, underscoring their essential roles in enhancing data recovery accuracy.
3. The study evaluated the data recovery capability of the PDO-BiGRU-GAN model under various missing-data scenarios. Results demonstrated that the model accurately reconstructed missing values by effectively leveraging underlying spatiotemporal dependencies, achieving an R^2^ greater than 0.93. Furthermore, to maintain optimal recovery performance, the missing data ratio within any given window should not exceed approximately 20/24.
4. The study compared the proposed PDO-BiGRU-GAN model with eight existing models in terms of accuracy and computational efficiency. Results indicated that PDO-BiGRU-GAN achieved the lowest values across all error metrics (MSE, RMSE, MAPE, MAE) and the highest R^2^, demonstrating a clear advantage in accuracy. Moreover, the model’s computation time increased only marginally. Overall, PDO-BiGRU-GAN substantially improved data recovery accuracy while maintaining efficient computational performance, highlighting its promise for pipeline monitoring applications.

## Data Availability

The original contributions presented in the study are included in the article/supplementary material, further inquiries can be directed to the corresponding author.

## Appendix 1: Pseudocode of the PDO model.

**Table:**

Initialization search_space = {learning_rate, batch_size, units, layers} pdo = PrairieDogOptimizationAlgorithm(search_space) best_params = pdo.optimize( fitness = function(params): generator = BiGRU_Generator(units = params.units, layers = params.layers) discriminator = BiGRU_Discriminator(units = params.units, layers = params.layers) gan = GAN (generator, discriminator, lr = params.learning_rate) gan.train(batch_size = params.batch_size, epochs = small_number) return gan.validation_loss() generator = BiGRU_Generator(units = best_params.units, layers = best_params.layers) discriminator = BiGRU_Discriminator(units = best_params.units, layers = best_params.layers) final_gan = GAN(generator, discriminator, lr = best_params.learning_rate) final_gan.train(batch_size = best_params.batch_size, epochs = full_training) recovered_data = final_gan.generate(missing_data) End

## References

[ref1] Abualigah L. Diabat A. Mirjalili S. Abd Elaziz M. Gandomi A. H. (2021). The arithmetic optimization algorithm. Comput. Methods Appl. Mech. Eng. 376:113609. doi: 10.1016/j.cma.2020.113609

[ref2] Adegboye M. A. Fung W. K. Karnik A. (2019). Recent advances in pipeline monitoring and oil leakage detection technologies: principles and approaches. Sensors 19:2548. doi: 10.3390/s19112548, PMID: 31167413 PMC6603558

[ref3] Agushaka J. O. Ezugwu A. E. Abualigah L. (2022). Dwarf mongoose optimization algorithm. Comput. Methods Appl. Mech. Eng. 391:114570. doi: 10.1016/j.cma.2022.114570

[ref4] Alqahtani H. Kavakli-Thorne M. Kumar G. (2021). Applications of generative adversarial networks (GANs): an updated review. Arch. Comput. Methods Eng. 28, 525–552. doi: 10.1007/s11831-019-09388-y

[ref5] Avula R. (2021). Addressing barriers in data collection, transmission, and security to optimize data availability in healthcare systems for improved clinical decision-making and analytics. Applied Research in Artificial Intelligence and Cloud Computing 4, 78–93.

[ref6] Bao X. Xu K. Liu J. Jin L. (2025). A physics-informed transformer model for long-sequence time-history response prediction of containment structures under mainshock-aftershock sequences. Eng. Struct. 343:121005. doi: 10.1016/j.engstruct.2025.121005

[ref7] Bian K. Priyadarshi R. (2024). Machine learning optimization techniques: a survey, classification, challenges, and future research issues. Arch. Comput. Methods Eng. 31, 4209–4233. doi: 10.1007/s11831-024-10110-w

[ref8] Biswas S. Shaikh A. Ezugwu A. E. S. Greeff J. Mirjalili S. Bera U. K.. (2024). Enhanced prairie dog optimization with levy flight and dynamic opposition-based learning for global optimization and engineering design problems. Neural Comput. & Applic. 36, 11137–11170. doi: 10.1007/s00521-024-09648-4

[ref9] Cai W. Wen X. Tu Q. Guo X. (2019). Research on image processing of intelligent building environment based on pattern recognition technology. J. Vis. Commun. Image Represent. 61, 141–148. doi: 10.1016/j.jvcir.2019.03.014

[ref10] Ciang C. C. Lee J. R. Bang H. J. (2008). Structural health monitoring for a wind turbine system: a review of damage detection methods. Meas. Sci. Technol. 19:122001. doi: 10.1088/0957-0233/19/12/122001

[ref11] DB32/T 2880-2016. (2016). Design, construction, and maintenance specifications for fiber optic sensing-based health monitoring systems for bridge and tunnel structures. Nanjing: Jiangsu Provincial Bureau of Quality and Technical Supervision. (In Chinese).

[ref12] Dohare S. Al Ansari M. S. Naga Ramesh J. V. El-Ebiary Y. A. B. Thenmozhi E. (2024). A hybrid GAN-BiGRU model enhanced by African buffalo optimization for diabetic retinopathy detection. Int. J. Adv. Comp. Sci. Appl. 15:970–81. doi: 10.3969/j.issn.1001-5256.2021.05.004

[ref13] Du J. Chen H. Zhang W. (2019). A deep learning method for data recovery in sensor networks using effective spatio-temporal correlation data. Sensor Rev. 39, 208–217. doi: 10.1108/SR-02-2018-0039

[ref14] Ezugwu A. E. Agushaka J. O. Abualigah L. Mirjalili S. Gandomi A. H. (2022). Prairie dog optimization algorithm. Neural Comput. & Applic. 34, 20017–20065. doi: 10.1007/s00521-022-07530-9

[ref15] Feng W. Q. Yin J. H. Borana L. Qin J.-Q. Wu P.-C. Yang J.-L. (2019). A network theory for BOTDA measurement of deformations of geotechnical structures and error analysis. Measurement 146, 618–627. doi: 10.1016/j.measurement.2019.07.010

[ref16] Gong X. Wang X. Li N. (2022). Research on DUAL-ADGAN model for anomaly detection method in time-series data. Comput. Intell. Neurosci. 2022, 1–18. doi: 10.1155/2022/8753323 PMC9629927 36337267

[ref17] Gu H. Wang T. Zhu Y. Wang C. Yang D. Huang L. (2021). A completion method for missing concrete dam deformation monitoring data pieces. Appl. Sci. 11:463. doi: 10.3390/app11010463

[ref19] Ho M. El-Borgi S. Patil D.. (2020). Inspection and monitoring systems subsea pipelines: a review paper. Struct. Health Monit. 19, 606–645. doi: 10.1177/1475921719837718

[ref20] Huang Y. Tang Y. VanZwieten J. Liu J. (2022). Reliable machine prognostic health management in the presence of missing data. Concurrency Computat. Pract. Exper. 34:e5762. doi: 10.1002/cpe.5762

[ref21] Izci D. Ekinci S. Hussien A. G. (2024). Efficient parameter extraction of photovoltaic models with a novel enhanced prairie dog optimization algorithm. Sci. Rep. 14:7945. doi: 10.1038/s41598-024-58503-y, PMID: 38575704 PMC10995185

[ref22] Jiang F. Ma J. Webster C. J. Li X. Gan V. J. L. (2023). Building layout generation using site-embedded GAN model. Autom. Constr. 151:104888. doi: 10.1016/j.autcon.2023.104888

[ref23] Jiang H. Wan C. Yang K. Ding Y. Xue S. (2022). Continuous missing data imputation with incomplete dataset by generative adversarial networks–based unsupervised learning for long-term bridge health monitoring. Struct. Health Monit. 21, 1093–1109. doi: 10.1177/14759217211021942

[ref24] Jieyang P. Kimmig A. Dongkun W. Niu Z. Zhi F. Jiahai W.. (2023). A systematic review of data-driven approaches to fault diagnosis and early warning. J. Intell. Manuf. 34, 3277–3304. doi: 10.1007/s10845-022-02020-0

[ref25] Kachuee M. Karkkainen K. Goldstein O. Darabi S. Sarrafzadeh M. (2020). Generative imputation and stochastic prediction. IEEE Trans. Pattern Anal. Mach. Intell. 44, 1278–1288. doi: 10.1109/TPAMI.2020.3022383 32894706

[ref26] Kennedy J. Eberhart R. (1995). “Particle swarm optimization” in Proceedings of ICNN'95-international conference on neural networks, (Perth, WA: IEEE), 4:1942–1948.

[ref27] Kosorukoff A. (2001). “Human based genetic algorithm” in 2001 IEEE international conference on systems, man and cybernetics, (Tucson: IEEE), 5:3464.

[ref28] Kumari R. Sarkar S. Dutta D.. (2024). “P2E-LGAN: PPG to ECG reconstruction methodology using LSTM-based generative adversarial network” in 2024 IEEE international symposium on circuits and systems (ISCAS). (Singapore: IEEE), 1–5.

[ref29] Kuntoğlu M. Salur E. Gupta M. K. Sarıkaya M. Pimenov D. Y. (2021). A state-of-the-art review on sensors and signal processing systems in mechanical machining processes. Int. J. Adv. Manuf. Technol. 116, 2711–2735. doi: 10.1007/s00170-021-07425-4

[ref30] Lei X. Siringoringo D. M. Sun Z. Fujino Y. (2023). Displacement response estimation of a cable-stayed bridge subjected to various loading conditions with one-dimensional residual convolutional autoencoder method. Struct. Health Monit. 22, 1790–1806. doi: 10.1177/14759217221116637

[ref31] Lei X. Sun L. Xia Y. (2021). Lost data reconstruction for structural health monitoring using deep convolutional generative adversarial networks. Struct. Health Monit. 20, 2069–2087. doi: 10.1177/1475921720959226

[ref32] Li Y. Sun Z. Mangalathu S. He W. Xue X. (2025a). Machine learning-based full-life-cycle seismic response assessment for in-service bridge piers: comprehensive analysis of interpretability and seismic fragility. Structure 80:110050. doi: 10.1016/j.istruc.2025.110050

[ref33] Li Y. Sun Z. Mangalathu S. Yang H. He W. (2025b). Seismic damage states prediction of in-service bridges using feature-enhanced swin transformer without reliance on damage indicators. Eng. Appl. Artif. Intell. 159:111651. doi: 10.1016/j.engappai.2025.111651

[ref34] Li P. Wang F. Gao J. Lin D. Gao J. Lu J.. (2022). Failure mode and the prevention and control technology of buried PE pipeline in service: state of the art and perspectives. Adv. Civ. Eng. 2022:2228690. doi: 10.1155/2022/2228690

[ref35] Li J. Wen M. Zhou Z. Wen B. Yu Z. Liang H.. (2024). Multi-objective optimization method for power supply and demand balance in new power systems. Int. J. Electr. Power Energy Syst. 161:110204. doi: 10.1016/j.ijepes.2024.110204

[ref36] Liu J. Liu J. Gao K. Mohagheghian I. Fan W. Yang J.. (2025a). A bioinspired gradient curved auxetic honeycombs with enhanced energy absorption. Int. J. Mech. Sci. 291:110189.

[ref37] Liu J. Zou Z. Gao K. Yang J. He S. Wu Z. (2025b). A novel digital unit cell library generation framework for topology optimization of multi-morphology lattice structures. Compos. Struct. 354:118824. doi: 10.1016/j.compstruct.2024.118824

[ref38] Liu J. Zou Z. Li Z. Zhang M. Yang J. Gao K.. (2025c). A clustering-based multiscale topology optimization framework for efficient design of porous composite structures. Comput. Methods Appl. Mech. Eng. 439:117881. doi: 10.1016/j.cma.2025.117881

[ref39] Lopez I. Sarigul-Klijn N. (2010). A review of uncertainty in flight vehicle structural damage monitoring, diagnosis and control: challenges and opportunities. Prog. Aerosp. Sci. 46, 247–273. doi: 10.1016/j.paerosci.2010.03.003

[ref40] Lv W. Sun Z. Li Y. Su L. He W. Zhang T. (2023). Hybrid machine learning-based model for predicting chloride ion concentration in coral aggregate concrete and its ethically aligned graphical user interface design. Mater Today Commun 37:107053. doi: 10.1016/j.mtcomm.2023.107053

[ref41] Mazumder R. K. Salman A. M. Li Y. Yu X. (2018). Performance evaluation of water distribution systems and asset management. J. Infrastruct. Syst. 24:03118001. doi: 10.1061/(ASCE)IS.1943-555X.0000426

[ref42] Mirjalili S. (2016). SCA: a sine cosine algorithm for solving optimization problems. Knowl.-Based Syst. 96, 120–133. doi: 10.1016/j.knosys.2015.12.022

[ref43] Mirjalili S. Gandomi A. H. Mirjalili S. Z. Saremi S. Faris H. Mirjalili S. M. (2017). Salp swarm algorithm: a bio-inspired optimizer for engineering design problems. Adv. Eng. Softw. 114, 163–191. doi: 10.1016/j.advengsoft.2017.07.002

[ref44] Mirjalili S. Mirjalili S. M. Lewis A. (2014). Grey wolf optimizer. Adv. Eng. Softw. 69, 46–61. doi: 10.1016/j.advengsoft.2013.12.007

[ref45] Noh S. H. (2021). Analysis of gradient vanishing of RNNs and performance comparison. Information 12:442. doi: 10.3390/info12110442

[ref46] Offiong N. M. Memon F. A. Wu Y. (2023). Time series data preparation for failure prediction in smart water taps (SWT). Sustainability 15:6083. doi: 10.3390/su15076083

[ref47] Oh E. Kim T. Ji Y. (2021). “STING: self-attention based time-series imputation networks using GAN” in 2021 IEEE international conference on data mining (ICDM) (Auckland, New Zealand: IEEE), 1264–1269.

[ref9001] Pu S. Li L. Xiang Y. Qiu X. (2022). Phase retrieval based on enhanced generator conditional generative adversarial network. in 2022 4th International Conference on Intelligent Control, Measurement and Signal Processing (ICMSP). IEEE, 825–829. doi: 10.1109/ICMSP55950.2022.9858954

[ref48] Quej-Ake L. M. Rivera-Olvera J. N. Domínguez-Aguilar Y. R. Avelino-Jiménez I. A. Garibay-Febles V. Zapata-Peñasco I.. (2020). Analysis of the physicochemical, mechanical, and electrochemical parameters and their impact on the internal and external SCC of carbon steel pipelines. Materials 13:5771. doi: 10.3390/ma13245771 33348736 PMC7766820

[ref49] Ren C. Xu Y. (2019). A fully data-driven method based on generative adversarial networks for power system dynamic security assessment with missing data. IEEE Trans. Power Syst. 34, 5044–5052. doi: 10.1109/TPWRS.2019.2922671

[ref50] Ressi D. Romanello R. Piazza C. Rossi S. (2024b). Ai-enhanced blockchain technology: a review of advancements and opportunities. J. Netw. Comput. Appl. 225:103858. doi: 10.1016/j.jnca.2024.103858

[ref51] Ressi D. Romanello R. Piazza C. Rossi S.. (2022). “Neural networks reduction via lumping” in International conference of the Italian Association for Artificial Intelligence (Cham: Springer International Publishing), 75–90.

[ref52] Ressi D. Romanello R. Rossi S. Piazza C. (2024a). Compressing neural networks via formal methods. Neural Netw. 178:106411. doi: 10.1016/j.neunet.2024.106411, PMID: 38906056

[ref53] Richter A. Ijaradar J. Wetzker U. Jain V. Frotzscher A. (2024). A survey on multivariate time series imputation using adversarial learning. IEEE Access 12, 148167–148189. doi: 10.1109/ACCESS.2024.3473540

[ref54] Schafer J. L. Graham J. W. (2002). Missing data: our view of the state of the art. Psychol. Methods 7, 147–177. doi: 10.1037/1082-989X.7.2.147 12090408

[ref55] Sharma V. B. Tewari S. Biswas S. Sharma A. (2024). A comprehensive study of techniques utilized for structural health monitoring of oil and gas pipelines. Struct. Health Monit. 23, 1816–1841. doi: 10.1177/14759217231183715

[ref56] Shen X. Zhao H. Xiang Y. Lan P. Liu J. (2022). Short-term electric vehicles charging load forecasting based on deep learning in low-quality data environments. Electr. Power Syst. Res. 212:108247. doi: 10.1016/j.epsr.2022.108247

[ref57] Shirazi H. Eadie R. Chen W. (2023). A review on current understanding of pipeline circumferential stress corrosion cracking in near-neutral pH environment. Eng. Fail. Anal. 148:107215. doi: 10.1016/j.engfailanal.2023.107215

[ref58] Siami-Namini S. Tavakoli N. Namin A. S. (2019). “The performance of LSTM and BiLSTM in forecasting time series” in 2019 IEEE international conference on big data (big data) (Los Angeles, CA, USA IEEE), 3285–3292.

[ref59] Silva L. O. Zárate L. E. (2014). A brief review of the main approaches for treatment of missing data. Intell. Data Anal. 18, 1177–1198. doi: 10.3233/IDA-140690

[ref60] Simon D. (2008). Biogeography-based optimization. IEEE Trans. Evol. Comput. 12, 702–713. doi: 10.1109/TEVC.2008.919004

[ref61] Sun Z. Li Y. Bei Y. Han T. Liu R. Wang L.. (2024c). Compressive strength resistance coefficient of sustainable concrete in sulfate environments: hybrid machine learning model and experimental verification. Mater Today Commun 39:108667. doi: 10.1016/j.mtcomm.2024.108667

[ref62] Sun Z. Li Y. Han T. Su L. Zhu X. He J.. (2024a). Performance evaluation of hybrid fiber-reinforced concrete based on electrical resistivity: experimental and data-driven method. Constr. Build. Mater. 446:137992. doi: 10.1016/j.conbuildmat.2024.137992

[ref63] Sun Z. Li Y. Li Y. Su L. He W. (2023a). Prediction of chloride ion concentration distribution in basalt-polypropylene fiber reinforced concrete based on optimized machine learning algorithm. Mater Today Commun 36:106565. doi: 10.1016/j.mtcomm.2023.106565

[ref64] Sun Z. Li Y. Li Y. Su L. He W. (2024b). Investigation on compressive strength of coral aggregate concrete: hybrid machine learning models and experimental validation. J. Build. Eng. 82:108220. doi: 10.1016/j.jobe.2023.108220

[ref65] Sun Z. Li Y. Su L. Liu S. Chen Z. (2025e). Predicting corrosion behaviour of steel reinforcement in eco-friendly coral aggregate concrete based on hybrid machine learning methods. Nondestruct. Test. Eval. 40, 1334–1354. doi: 10.1080/10589759.2024.2349243

[ref66] Sun Z. Li Y. Su L. Niu D. Luo D. He W.. (2024e). Investigation of electrical resistivity for fiber-reinforced coral aggregate concrete. Constr. Build. Mater. 414:135011. doi: 10.1016/j.conbuildmat.2024.135011

[ref67] Sun Z. Li Y. Yang Y. Su L. Xie S. (2024d). Splitting tensile strength of basalt fiber reinforced coral aggregate concrete: optimized XGBoost models and experimental validation. Constr. Build. Mater. 416:135133. doi: 10.1016/j.conbuildmat.2024.135133

[ref68] Sun Z. Niu D. Luo D. Wang X. Zhang L. Su L.. (2023b). Hybrid machine learning-based prediction model for the bond strength of corroded Cr alloy-reinforced coral aggregate concrete. Mater Today Commun 35:106141. doi: 10.1016/j.mtcomm.2023.106141

[ref69] Sun Z. Wang X. Han T. Huang H. Ding J. Wang L.. (2025a). Pipeline deformation monitoring based on long-gauge fiber-optic sensing systems: methods, experiments, and engineering applications. Measurement 248:116911. doi: 10.1016/j.measurement.2025.116911

[ref70] Sun Z. Wang X. Han T. Huang H. Huang X. Wang L.. (2025c). Pipeline deformation monitoring based on long-gauge FBG sensing system: missing data recovery and deformation calculation. J. Civ. Struct. Heal. Monit. 15, 2433–2453. doi: 10.1007/s13349-025-00943-9

[ref71] Sun Z. Wang X. Han T. Wang L. Zhu Z. Huang H.. (2025b). Pipeline deformation prediction based on multi-source monitoring information and novel data-driven model. Eng. Struct. 337:120461. doi: 10.1016/j.engstruct.2025.120461

[ref72] Sun Z. Wang X. Huang H. Yang Y. Wu Z. (2024f). Predicting compressive strength of fiber-reinforced coral aggregate concrete: interpretable optimized XGBoost model and experimental validation. Structure 64:106516. doi: 10.1016/j.istruc.2024.106516

[ref73] Sun Z. Wang X. Niu D. Luo D. Han T. Li Y.. (2025d). Electrical resistivity prediction model for basalt fibre reinforced concrete: hybrid machine learning model and experimental validation. Mater. Struct. 58, 1–22. doi: 10.1617/s11527-025-02607-y

[ref74] Taheri S. M. Hesamian G. (2013). A generalization of the Wilcoxon signed-rank test and its applications. Stat. Pap. 54, 457–470. doi: 10.1007/s00362-012-0443-4

[ref75] Tan S. Song W. Z. Stewart M. Yang J. Tong L. (2016). Online data integrity attacks against real-time electrical market in smart grid. IEEE Trans. Smart Grid 9, 313–322. doi: 10.1109/TSG.2016.2550801

[ref76] Tien T. B. Quang T. V. Ngoc L. N. Bui Tien T. Vu Quang T. Nguyen Ngoc L.. (2024). Time series data recovery in SHM of large-scale bridges: leveraging GAN and bi-LSTM networks. Structure 63:106368. doi: 10.1016/j.istruc.2024.106368

[ref77] Torres B. Payá-Zaforteza I. Calderón P. A. Adam J. M. (2011). Analysis of the strain transfer in a new FBG sensor for structural health monitoring. Eng. Struct. 33, 539–548. doi: 10.1016/j.engstruct.2010.11.012

[ref78] TSG D7005-2018. (2018). Periodic inspection regulations for pressure pipelines – industrial pipelines. Beijing: General Administration of Quality Supervision, Inspection and Quarantine of the People’s Republic of China. (In Chinese).

[ref79] Vasagar V. Hassan M. K. Abdullah A. M. Karre A. V. Chen B. Kim K.. (2024). Non-destructive techniques for corrosion detection: a review. Corros. Eng. Sci. Technol. 59, 56–85. doi: 10.1177/1478422X241229621

[ref80] Wan Q. Liu J. (2023). Energy efficiency optimization and carbon emission reduction targets of resource-based cities based on BiLSTM-CNN-GAN model. Front. Ecol. Evol. 11:1248426. doi: 10.3389/fevo.2023.1248426

[ref82] Wang B. Zeng Y. Feng D. (2025a). Deep learning-based damage assessment of hinge joints for multi-girder bridges utilizing vehicle-induced bridge responses. Eng. Struct. 333:120148. doi: 10.1016/j.engstruct.2025.120148

[ref83] Wang B. Zeng Y. Feng D. Li J.-a. (2025b). Track vertical irregularity estimation for railway bridges using a novel and lightweight deep-learning architecture. Veh. Syst. Dyn. 63, 1597–1624. doi: 10.1080/00423114.2024.2375031

[ref81] Wang K. Shen T. Wei J. Liu J. Hu W. (2025c). An intelligent framework for deriving formulas of aerodynamic forces between high-rise buildings under interference effects using symbolic regression algorithms. J. Build. Eng. 99:111614. doi: 10.1016/j.jobe.2024.111614

[ref84] Wei J. Shen T. Wang K. Liu J. Wang S. Hu W. (2025). Transfer learning framework for the wind pressure prediction of high-rise building surfaces using wind tunnel experiments and machine learning. Build. Environ. 271:112620. doi: 10.1016/j.buildenv.2025.112620

[ref85] Wong B. McCann J. A. (2021). Failure detection methods for pipeline networks: from acoustic sensing to cyber-physical systems. Sensors 21:4959. doi: 10.3390/s21154959, PMID: 34372194 PMC8348533

[ref86] Woolson R. F. (2007). Wilcoxon signed-rank test. in Wiley Encyclopedia of Clinical Trials, (Hoboken, NJ: John Wiley & Sons). 1–3.

[ref87] Wright R. F. Lu P. Devkota J. Lu F. Ziomek-Moroz M. Ohodnicki Jr P. R.. (2019). Corrosion sensors for structural health monitoring of oil and natural gas infrastructure: a review. Sensors 19:3964. doi: 10.3390/s19183964 31540327 PMC6767297

[ref88] Wu T. Liu G. Fu S. Xing F. (2020). Recent progress of fiber-optic sensors for the structural health monitoring of civil infrastructure. Sensors 20:4517. doi: 10.3390/s20164517, PMID: 32806746 PMC7472180

[ref89] Wu Z. Ma C. Shi X. Wu L. Zhang D. Tang Y.. (2021). “BRNN-GAN: generative adversarial networks with bi-directional recurrent neural networks for multivariate time series imputation” in 2021 IEEE 27th international conference on parallel and distributed systems (ICPADS). (Beijing: IEEE), 217–224.

[ref90] Xie S. Lin H. Chen Y. Yao R. Sun Z. Zhou X. (2025a). Hybrid machine learning models to predict the shear strength of discontinuities with different joint wall compressive strength. Nondestruct. Test. Eval. 40, 2439–2459. doi: 10.1080/10589759.2024.2381083

[ref91] Xie S. Lin H. Ma T. Peng K. Sun Z. (2025b). Prediction of joint roughness coefficient via hybrid machine learning model combined with principal components analysis. J. Rock Mech. Geotech. Eng. 17, 2291–2306. doi: 10.1016/j.jrmge.2024.05.059

[ref92] Xiong J. Zhu J. He Y. Ren S. Huang W. Lu F. (2020). The application of life cycle assessment for the optimization of pipe materials of building water supply and drainage system. Sustain. Cities Soc. 60:102267. doi: 10.1016/j.scs.2020.102267

[ref93] Xu Q. Zhang L. Liang W. (2013). Acoustic detection technology for gas pipeline leakage. Process. Saf. Environ. Prot. 91, 253–261. doi: 10.1016/j.psep.2012.05.012

[ref94] Yang Z. Yang D. Dyer C. He X. Smola A. Hovy E.. (2016). Hierarchical attention networks for document classification. In Proceedings of the 2016 Conference of the North American Chapter of the Association for Computational Linguistics: Human Language Technologies. (Austin, TX: Association for Computational Linguistics), 1480–1489.

[ref9002] Zhang Q. Wang T. (2024). Deep learning for exploring landslides with remote sensing and geo-environmental data: Frameworks, progress, challenges, and opportunities. Remote Sens 16, 1344. doi: 10.3390/rs16081344

[ref95] Zhou J. (2023). Visualization of green building landscape space environment design based on image processing and artificial intelligence algorithm. Soft. Comput. 27, 10225–10235. doi: 10.1007/s00500-023-08266-x

